# A cluster analysis of neonates using clinical signs of possible serious bacterial infection at hospital admission in Kenya: A retrospective multicentre cohort study

**DOI:** 10.1371/journal.pone.0359212

**Published:** 2026-09-24

**Authors:** Timothy Tuti, Tabitha Muema, Mike English, on behalf of The Clinical Information Network Group, Jalemba Aluvaala

**Affiliations:** 1 KEMRI-Wellcome Trust Research Program, Nairobi, Kenya; 2 Centre for Tropical Medicine and Global Health, Nuffield Department of Medicine, University of Oxford, Oxford, United Kingdom; 3 Health Systems Collaborative, Nuffield Department of Medicine, University of Oxford, Oxford, United Kingdom; 4 Department of Paediatrics and Child Health, University of Nairobi, Kenyatta National Hospital Nairobi, Nairobi, Kenya; Freelance Consultant, Myanmar, MYANMAR

## Abstract

**Background:**

Neonatal sepsis remains a major cause of mortality in Sub-Saharan Africa (SSA). Despite presenting with considerable clinical heterogeneity, suspected cases are managed uniformly with broad-spectrum antibiotics. Typical data-driven approaches developed in high-resource settings to identify clinically meaningful phenotypes and support management of neonatal sepsis have limited generalisability to many SSA public hospital settings, due to inclusion of variables that are largely unavailable at admission. This study’s objective was to identify sepsis clusters using signs of possible Serious Bacterial Infection (pSBI) readily available at the time of admission, and to assess the clusters performance in predicting mortality.

**Methods:**

We conducted unsupervised model-based cluster analysis using Latent Class Analysis based on pSBI data collected at admission. All neonates <28 days old admitted to 21 Kenyan hospitals between January 2022 and December 2024 with ≥1 pSBI sign/symptom at admission were eligible for inclusion. We further explored the external validity of this clustering approach on new patient populations, and assessed the ability of the identified clusters to accurately predict in-hospital mortality compared to the World Health Organization neonatal sepsis severity classification guidelines.

**Results:**

Five clusters of minimal, low, moderate, substantial and critical mortality risk were identified from development dataset with 33094 patients from eight hospitals. The models had an accuracy, positive predictive value and specificity of at least 83.16% (82.72% to 83.62%), 81.02% (80.58% to 81.45%) and 86.91% (86.61% to 87.23%) respectively in predicting cluster membership of 23704 patients in the external validation dataset admitted to thirteen different hospitals. From an internal-external cross-validation approach of the in-hospital mortality risk, the model-based clustering approach had discrimination (AUROC) of 0.867 (0.863 to 0.871) and calibration intercept and slope of −0.004 (−0.031 to 0.023) and 0.996 (0.979 to 1.014) respectively, outperforming the WHO sepsis severity classification whose discrimination was 0.721 (0.715 to 0.727) and calibration intercept and slope being 0.018 (−0.005 to 0.041) and 1.015 (0.986 to 1.043) respectively.

**Conclusion:**

The identified clusters can complement clinicians’ judgement in assessing risk among neonates with sepsis at admission. Future work evaluating the utility of these clusters and potential differences in treatment response across clusters are therefore recommended to help strengthen the case for more targeted, risk-based neonatal sepsis management.

## Background

The most vulnerable time for a child’s survival are the first 28 days of life (the neonatal period), with 2.3 million children dying during this period globally, with a child from Sub-Saharan Africa (SSA) being 10 times more likely to die than a child from a high-income country [1]. Sepsis, which is a dysregulated immune response to infection that leads to acute organ dysfunction, is a leading contributor to burden of disease in neonates in SSA, both as primary cause of death and as a frequent contributor [2,3], and carries a high risk of death even when care is provided promptly [4].

In newborns, sepsis occurs in two forms: early-onset neonatal sepsis, which occurs within the first 72 hours of life, often linked to complications during birth, and late-onset neonatal sepsis, which develops between after 72 hours of life [5]. These infections are more common in premature or low birth weight babies, and their rates are higher in regions with limited or poor quality healthcare [6]. One-sixth of neonates treated for sepsis face significant challenges including functional limitation, cognitive impairment, and mental health disorders after recovery with 40% of sepsis patients requiring rehospitalisation within 90 days of discharge [3].

Many of these neonatal sepsis deaths in SSA are preventable with early detection and proper treatment. To achieve this healthcare providers rely on a high index of suspicion of sepsis (in the absence of laboratory confirmation) based on signs of possible Serious Bacterial Infection (pSBI) like poor feeding, lethargy, temperature instability, or respiratory distress [7,8]. While laboratory tests such as blood cultures to identify pathogens, complete blood counts to assess the immune response, and urine cultures for accurate sepsis diagnosis are crucial for effective treatment [9], in many health facilities in SSA, these diagnostic tests are unavailable at the time of admission [10].

There is no consensus on the clinical definition of neonatal sepsis where microbiological confirmations are unavailable [11,12]. It is a heterogeneous syndrome representing a diverse group of patients, ranging from neonates with minor infections that will resolve quickly to those with severe life-threatening sepsis. Using broad-spectrum, empiric antibiotic treatment for all suspected cases of neonatal sepsis may not be ideal, as it can lead to unnecessary prolonged antibiotic use in non-infected infants, potentially causing harm [4]. Tailoring treatment based on specific diagnoses and risk of mortality could improve outcomes and reduce overuse of antibiotics [13].

The challenge lies in developing alternative effective strategies for early diagnosis and treatment of neonatal sepsis in environments such as SSA where microbiological investigation or laboratory testing is either unavailable or inaccessible [10]. Different combinations of pSBI signs may naturally cluster into previously undescribed subsets or phenotypes that may have different risks for the outcome and may respond differently to treatments. Identification of distinct clinical phenotypes may allow more precise therapy and improve care [4]. However, many guidelines and hospital protocols continue to recommend a one-size-fits-all approach; recommending the same initial inpatient treatment and follow up [4].

Efforts to address the variability in neonatal sepsis have included the WHO’s triaging guidelines for pSBI [4,14] as illustrated in S1 Table. Other approaches have included use of (1) cluster analysis of neonates based on clinical and laboratory data from electronic health records (EHRs) collected within the first six hours of the patients’ hospital stay, combined with serum biomarkers [15], (2) latent class analysis (LCA) to identify molecular phenotypes in sepsis patients using both clinical and protein biomarker data [16], and even used self-organizing maps to identify sepsis groups susceptible to multiple organ dysfunction syndrome based on their age and sequential organ failure assessment scores [17].

However, these approaches generally rely on laboratory, biomarker, or other variables that are not routinely available at admission in many SSA health facilities, limiting their applicability in these settings [4,10,18]. Moreover, there is limited evidence on whether clinically derived phenotypes based on routinely collected admission data are reproducible across hospitals or can be translated into simple classifiers for assigning new patients to predefined risk groups [19]. Addressing these gaps could provide a more context-appropriate approach to neonatal risk stratification that is both externally valid and feasible for routine use in resource-constrained settings [18,19].

Using routinely collected data that is available in typical SSA hospital settings, the aim of this study is to identify clusters of neonates at time of admission that can be candidates for appropriate interventions to maximise patient outcomes given the resource constraints. More specifically, the objectives of this study were:

1. To use unsupervised model-based LCA clustering approach to group and assign neonates into clinically meaningful clusters based on key clinical features (i.e. pSBI) from data routinely collected at admission in many SSA health facilities.
2. To explore the external validity and transportability of the model-based clustering approach to different patient populations in similar clinical contexts through:
  a) Comparing clustering performance when the LCA model fitted on the development dataset is applied to an external dataset (i.e., data from new hospitals) versus when a new LCA model is fitted directly to the external dataset.
  b) Evaluating the accuracy of a parsimonious classification model for assigning new patients to the clusters identified by the LCA model in objective 1.
3. To assesses the identified clusters ability to predict in-hospital mortality and compare this with the World Health Organization (WHO) guideline-based approach to neonatal sepsis severity classification.

## Methods

### Ethics and reporting

The reporting of this study follows the Strengthening the Reporting of Observational Studies in Epidemiology (STROBE) guidelines, which provide a set of recommendations for transparent and comprehensive reporting of observational studies using cohort, case-control, or cross-sectional designs [20]. The reporting also follows the Transparent Reporting of a multivariable prediction model for Individual Prognosis Or Diagnosis (TRIPOD) guidelines, which is a set of recommendations for the reporting of studies developing, validating, or updating prediction models for prognostic purposes [21].

The Scientific and Ethics Review Unit of the Kenya Medical Research Institute (KEMRI) approved the collection of a copy of the de-identified anonymised patient-level data that provides the basis for this study as part of the Clinical Information Network (CIN) which runs in partnership with the Ministry of Health and the participating hospitals (SERU #3459). We elaborate on the CIN in the study design and settings section. Individual consent for retrospective access to the de-identified anonymised patient records was not required and was waived by KEMRI with the authors having no access to the information that could identify the patients.

### Study design and participants

This is a retrospective cohort analysis utilizing data from the Clinical Information Network (CIN). The CIN collects standardised routine admission and discharge data from newborn units (NBU) across 21 public county hospitals in 14 out of the 47 counties in Kenya with a detailed description of the methods of data collection and management is provided elsewhere [22]. In brief, neonatal admission data are recorded using paper forms such as Neonatal Admission Records (NAR), treatment sheets, continuous monitoring charts, supplementary forms etc. These documents provide a structured checklist for typically junior clinicians [23], covering nine essential domains: demographics, admission details, maternal history, presenting complaints, cardinal signs, physical examinations, nursing monitoring, discharge status and supportive care. The hospital forms are designed to record the clinical data outlined in the **“**Predictors**”** subsection of the Methods in accordance with WHO and national clinical guidelines, thereby supporting evidence-based clinical decision-making [14,24]. Consequently, the recorded variables correspond directly to the relevant WHO criteria without requiring additional mapping. This approach to clinical form design and data collection is widely used across East and Southern Africa [25]. Each hospital has a clerk who then extracts data from these typical hospital forms into a Research Electronic Data Capture (REDCap) database immediately after patient death or discharge [26].

The study participants included inborn neonates aged 0–28 days admitted into newborn units between 01^st^ January 2022 and 31^st^ December 2024 with at least one pSBI sign/symptom at admission, regardless of whether they had a neonatal sepsis admission diagnosis, or were prescribed first-line intravenous antibiotics (e.g., Crystalline Penicillin and Gentamicin) at admission [27]. Data from 8 hospitals (n = 33094) was used as model derivation dataset, with the external geographical validation dataset coming from 13 hospitals (n = 23704); Hospitals in the derivation dataset represent large hospitals with ≥1000 NBU admissions per year, with the validation dataset having hospitals with <1000 NBU admissions per year. Although hospital volume may capture differences in patient populations and care settings, it was used as a pragmatic criterion to define the development and validation datasets to ensure that the development dataset contained a sufficiently large number of neonates to support stable estimation of the model-based clusters while minimising the influence of very small hospital-specific samples.

### Outcome

The primary objective of this study is to identify clinically meaningful sub-populations of neonates with pSBI signs at admission based on key clinical features, derived using unsupervised clustering techniques fitted on the development dataset (n = 33094 admissions from 8 hospitals)**.** The selected pSBI clinical variables included signs of local infection, fever, hypothermia, difficulty feeding, difficulty breathing, convulsions, apnoea, floppy, indrawing, grunting, crackles, jaundice, slow capillary refill, central cyanosis, irritable, hypoxic, and bulging fontanelle [14]. The second objective’s outcome was the predicted cluster membership in the external dataset (n = 23704 admissions from 13 hospitals) from a parsimonious classification model compared to the derived cluster membership from using the original model-based clustering approach from objective 1. For the third objective, the outcome of interest was mortality at discharge.

### Predictors

This study analysed two sets of predictors corresponding to the study objectives. For the first and second objective, clinical variables listed in the section above which are commonly associated with sepsis/pSBI [14] were used to identify and characterise distinct clusters of neonates. Additionally, objective two adopted feature selection based on feature importance to identify a parsimonious set of pSBI predictors for cluster membership classification [28].

The identified clusters from objective one are the primary predictor variables for objective three, when assessing their association with mortality outcome; To ensure robust estimation, adjustments were made for potential confounders, neonate’s age and birth weight, given their well-established impact on length of stay and mortality [29–31].

### Missing data

Previous studies using CIN data showed that missing at random (MAR) is a reasonable assumption for our dataset [32]; For objective one, the model-based clustering approach adopts a Full Information Maximum Likelihood (FIML) approach where the cluster memberships are computed using all available information while taking the missingness into account under the MAR assumption, therefore no imputation was required [33].

For objective three, with the MAR assumption, multiple imputation using the chained equation approach was separately applied for both the development and validation datasets [34] in predicting in-hospital mortality outcome.

### Statistical analysis methods

#### Clustering approaches for neonates with pSBI managed for sepsis.

Latent Class Analysis (LCA) which is a Finite Mixture Modelling approach was used for objective one since it offered a model-based (i.e., probabilistic) approach to derive clusters [33,35]; LCA adopts Full Information Maximum Likelihood (FIML) approach making use of observations with missing data in the cluster estimation process [33,35,36]. LCA uses the distribution of the dataset to assess probabilities that certain patients are members of certain clusters. Best model-fit was determined by evaluating different metrics from LCA approach summarised in S2 Table; In summary the most probable number of clusters is determined by clear delineation (i.e., entropy thresholds >0.8), the smallest cluster size having considerable number of admissions, with the cluster model able to accurately predicting class membership for individual patients (i.e., Average Latent Class Posterior Probability (ALCPP) thresholds >0.85) (S2 Table). The final LCA model used to generate the clusters in the development dataset was also used to also predict the cluster membership of the validation dataset.

For each LCA-derived cluster, the label was assigned by the research team after examining the mortality outcome and the cluster’s characteristic pattern of conditional response probabilities across the observed clinical variables; and assigning a clinically meaningful descriptor that reflected its dominant features [37].

As part of sensitivity analysis, we compared the model-based clustering approach to a distance-based unsupervised machine learning technique of agglomerative hierarchical cluster analysis (HCA) that groups patients with similar pSBI into clusters without imposing a specific sequential order on them [38,39]. Agglomerative HCA was selected for sensitivity analysis due to its efficiency in handling mixed data types and its ability to generate clinically meaningful clusters that are stable and easy to interpret [40]. Given our dataset consists of both continuous and categorical variables, standard Euclidean distance measures suited for numerical data, were unsuitable [41]; Gower’s distance which is well suited for mixed data types was used instead [42,43], together with Ward’s minimum variance method as the linkage criterion to group neonates into meaningful clusters by minimising the total within-cluster variance [44]. The optimal number of clusters from HCA approach was determined using the silhouette score [45]. The Silhouette Score evaluated how well individual data points fit within their assigned clusters relative to the nearest alternative cluster, and ranges from −1–1 with higher scores indicating better clustering [45].

#### External validation and transportability of the clustering model.

For model external validation in objective 2(a), the predicted class-membership from the original fitted LCA model in objective 1 was compared to class-membership from a new LCA model fitted directly to the external dataset.

For objective 2(b), we used eXtreme Gradient Boosting (xgboost), a machine learning technique with implicit features selection that is robust to missing data patterns, to identify a subset of pSBI features based on feature importance to predict cluster membership [46,47]. Feature selection was based on SHAP (SHapley Additive exPlanations) values where the SHAP values derived from xgboost model which explain how the patient-level variables contribute to the cluster assignment [48]; The process is explained in detail in S1 Text. Variables for inclusion in the final model were selected by visual inspection (“eyeballing”) of the SHAP summary plots, prioritising predictors that demonstrated relatively large and consistent absolute SHAP contributions across observations. The parsimonious multivariate logistic regression model was developed from the development dataset (n = 33094 admissions from 8 hospitals).

For evaluation of model transportability in objective 2(b) using the validation dataset (n = 23704 admissions from 13 hospitals), the predicted class-membership from the parsimonious model (which was determined based on which cluster the patient had the highest probability of being a member of), was compared to class-membership of the validation dataset predicted from the original fitted LCA model in objective one.

Typical classification metrics of accuracy, sensitivity, specificity, positive predictive value (PPV) etc. were used to report performance of the parsimonious classification model to correctly cluster patients into the model-based clusters.

#### Logistic regression for in-hospital mortality.

For objective three, logistic regression without variable selection was used together with the imputed datasets with parameter estimates being combined using Rubin’s rule. The number of imputation datasets to use was determined from the integer value of the percentage of patients in the derivation dataset that had one or more missing values, rounded upwards. To examine heterogeneity in model performance while incorporating geographical external validation, we compared the logistic regression models internal-external cross-validation performance where we omitted one hospital at a time using it as the validation dataset, built the model on the remaining hospitals, and evaluated model’s discrimination and calibration performance on the hospital left out. We repeated this process with each iteration using a different hospital as the validation data source [49]. The predictive performance of in-hospital mortality by the model-based and hierarchical clustering approaches was then compared to the WHO’s pSBI guideline-based clustering approach (S1 Table). Model performance was assessed using calibration and discrimination performance metrics detailed in S3 Table, with the confidence intervals for both c-statistic and calibration slope and intercept, calculated through 1000 iterative bootstrap samples with replacement, by resampling 10% of the held-out hospital dataset (i.e., the validation dataset) in each iteration.

### Sample size

For the first and second objective, given clustering is an unsupervised statistical learning technique that does not rely on predefined sample sizes but rather extracted patterns from the available data [50,51], coupled with a large development dataset (n = 33094 admissions) and validation dataset (n = 23704 admissions), a formal sample size calculation was deemed unnecessary. All eligible neonatal records were thus included to maximize the robustness of the clustering approach.

For the third objective, we based our sample size calculation on previously reported mortality outcome prevalence of between 9% and 14% with R-squared values of 0.453 derived from previously-developed mortality risk prediction models in similar neonatal patient populations [52]. Using the pmsampsize library in R [53] and assuming a maximum of six clinically-meaningfully clusters identified [54,55] after including age (in days) and birth weight, the required sample size for the mortality risk model development and validation would be 260 patients with 24 deaths; making both our development dataset with 33094 admissions (with 9768 deaths) and validation dataset with 23704 admissions (with 4180 deaths) satisfactory for in-hospital mortality clinical prediction model development and external validation (S4 Table).

## Results

### Description of participants

**Fig 1:**
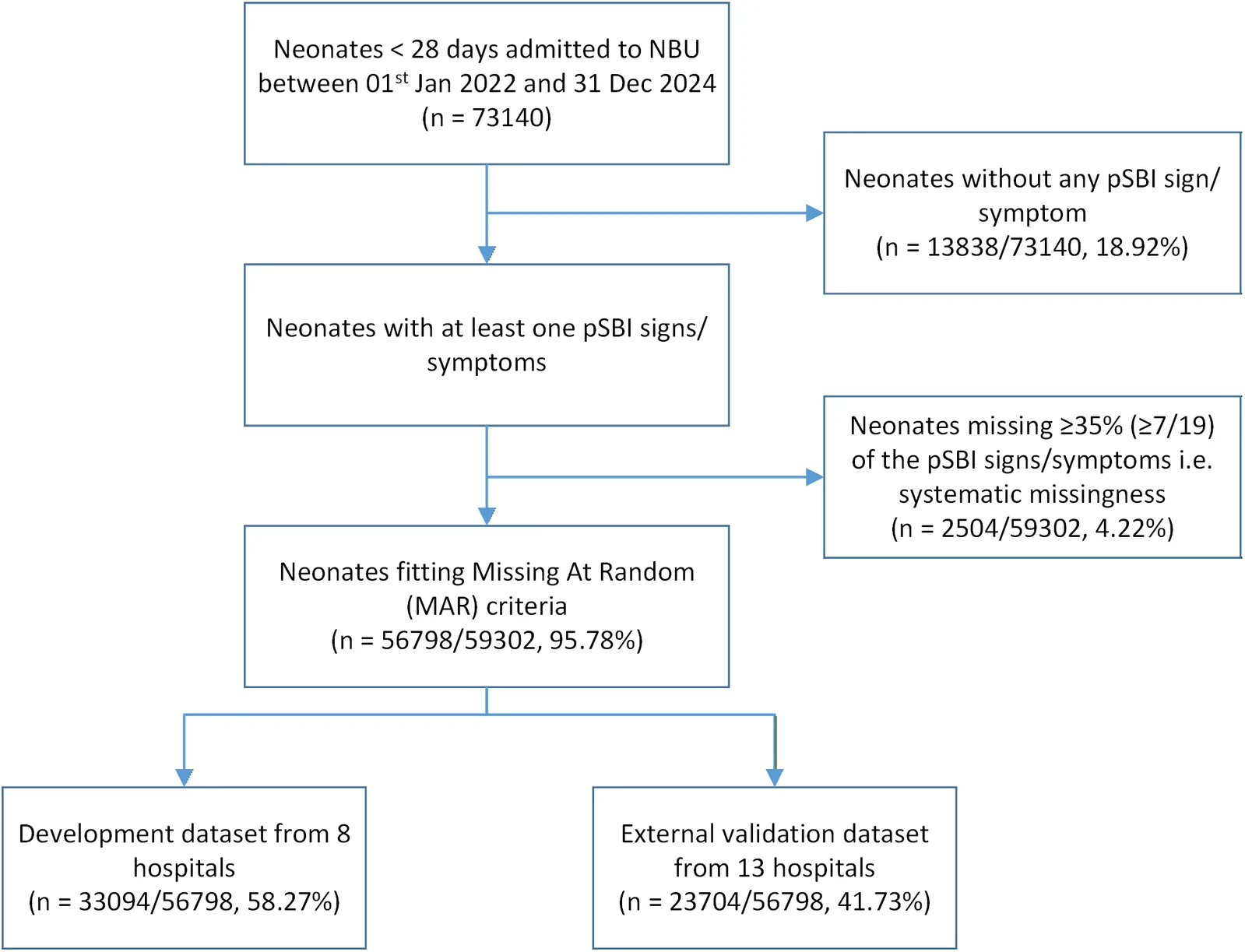
Patients included in analyses grouped by development and external validation dataset.

Between 01^st^ January 2022 and 31^st^ December 2024, 73140 neonates aged 0–28 days were admitted to newborn units across the 21 CIN hospitals. For analysis, 59302/73140 (81.08%) of the admissions had at least one pSBI sign/symptom and were eligible for inclusion in the study (Fig 1) with patterns of pSBI signs/symptoms illustrated in Table 1 and S1 Fig; Of the 59302 admissions with ≥1 pSBI sign/symptom, 2504/59302 (4.22%) had signs of systematic missingness (i.e., ≥ 7/19 (≥35%) pSBI signs not documented; Within the CIN, this level of missingness is indicative of missing documentation due to patients with either severe symptoms, in emergency situations or paper documents removed from patient files (S2 Fig); The patients with systematic missingness were excluded due to high likelihood of introducing bias if included in subsequent analysis due to violating the missing at random (MAR) assumption. The neonatal admissions in CIN during this period were divided geographically by hospitals into a development dataset from 8 CIN hospitals (n = 33094/56798, 58.27%), and a validation set from the remaining 13 CIN hospitals (n = 23704/56798, 41.73%) (Table 1). Excluding sepsis admission diagnoses, respiratory distress syndrome, birth asphyxia, and meconium aspiration were the most common admission diagnoses in the included patients (S3 Fig).

**Table 1:** Baseline characteristics of patients included in the analyses.

Indicator	Development Dataset (n = 33094)		Validation Dataset (n = 23704)	
	Summary Statistic	Missing Rate	Summary Statistic	Missing Rate
Male	18935 (57.22%)	48 (0.15%)	13198 (55.68%)	67 (0.28%)
Age (in days)	1 (IQR: 1–1)	0 (0%)	1 (IQR: 1–1)	0 (0%)
Birth weight (in kgs)	2.65 (IQR: 1.86 to 3.2)	0 (0%)	2.7 (IQR: 1.81 to 3.2)	0 (0%)
Gestational age (in weeks)	38 (IQR: 34–39)	2474 (7.48%)	38 (IQR: 33–39)	1851 (7.81%)
Length of Stay (in days)	5 (IQR: 3–11)	0 (0%)	5 (IQR: 3–11)	0 (0%)
Number of pSBI signs/ symptoms	3 (IQR: 1–4)	0 (0%)	3 (IQR: 2–4)	0 (0%)
Apnoea	1560 (4.71%)	646 (1.95%)	1677 (7.07%)	249 (1.05%)
History of fever	2935 (8.87%)	761 (2.3%)	3779 (15.94%)	435 (1.84%)
Abnormal temperature^a^	4518 (13.65%)	3162 (9.55%)	5164 (21.79%)	2003 (8.45%)
Difficulty feeding	9500 (28.71%)	1000 (3.02%)	6969 (29.4%)	819 (3.46%)
Difficulty breathing	18309 (55.32%)	598 (1.81%)	11275 (47.57%)	271 (1.14%)
Convulsions	1905 (5.76%)	564 (1.7%)	2092 (8.83%)	189 (0.8%)
Floppy	17731 (53.58%)	2450 (7.4%)	11051 (46.62%)	2636 (11.12%)
Tachypnoea^b^	12151 (36.72%)	4056 (12.26%)	7955 (33.56%)	3050 (12.87%)
Indrawing	3792 (11.46%)	664 (2.01%)	2006 (8.46%)	605 (2.55%)
Local infection	354 (1.07%)	798 (2.41%)	541 (2.28%)	311 (1.31%)
Grunting	6304 (19.05%)	299 (0.9%)	3310 (13.96%)	490 (2.07%)
Crackles	2093 (6.32%)	421 (1.27%)	1549 (6.53%)	632 (2.67%)
Jaundice	1777 (5.37%)	964 (2.91%)	2026 (8.55%)	839 (3.54%)
Slow capillary refill^c^	2085 (6.3%)	4495 (13.58%)	1916 (8.08%)	1349 (5.69%)
Central cyanosis	1560 (4.71%)	642 (1.94%)	1089 (4.59%)	632 (2.67%)
Irritable	2300 (6.95%)	580 (1.75%)	2383 (10.05%)	471 (1.99%)
Hypoxic^d^	6071 (18.34%)	3260 (9.85%)	4133 (17.44%)	2949 (12.44%)
Bulging fontanelle	442 (1.34%)	544 (1.64%)	454 (1.92%)	360 (1.52%)
Abnormal heart rate^e^	1518 (4.59%)	3144 (9.5%)	1571 (6.63%)	2131 (8.99%)
Died	5588 (16.89%)	0 (0%)	4180 (17.63%)	0 (0%)

^a^Temparature ≥ 38.0°C or ≤35.5°C.

^a^Respiratory rate ≥ 60 breaths per minute.

^c^Capillary refill  ≥ 3 seconds.

^c^Pulse oximetry < 90%.

^e^Heart rate <100 or >159 beats per minute.

### Findings from latent class analysis

Table 2 illustrates the model-based clustering fit statistics in the development dataset. The identified clusters are of patients with pSBI managed for sepsis grouped to maximise similarity in pSBI characteristics. From the model fit statistics described in S2 Table, the maximum number of clusters with clear delineation (i.e., entropy thresholds > 0.8), whose smallest cluster size had arguably considerable number of admissions, and whose cluster model was able to accurately predicting class membership for individual patients (i.e., Average Latent Class Posterior Probability (ALCPP) thresholds > 0.85), is five (Table 2).

**Table 2:** Model fit indices from Latent Class Analysis Fit Indices.

Clusters^*a*^	Parameters	LL^*b*^	AIC^*c*^	BIC^*d*^	aBIC^*e*^	Entropy^f^	Smallest Cluster Size	ALCPP^*g*^	LMR P-value^*h*^
2	53	−367766	735639	736084	735916	0.870	10792 (32.61%)	0.957	<0.001
*3*	*79*	*−360415*	*720989*	*721653*	*721402*	*0.785*	*7162 (21.64%)*	*0.897*	*<0.001*
*4*	*105*	*−357058*	*714327*	*715210*	*714876*	*0.752*	*4551 (13.75%)*	*0.857*	*<0.001*
5	131	−351102	702466	703568	703151	0.803	3368 (10.18%)	0.883	<0.001
6	157	−350771	701857	703177	702678	0.799	1754 (5.3%)	0.844	<0.001

^a^Clustering mixture model performance metrics from MPLUS 7.1 software.

^b^Log-Likelihood.

^c^Akaike Information Criteria (AIC).

^d^Bayesian Information Criteria (BIC).

^e^Sample-Size adjusted Bayesian Information Criteria (aBIC).

^f^How clear the class delineation is; How well the classes separate the population into distinct subgroups of patients.

^g^Average Latent Class Posterior Probability (ALCPP). The average probability of the class model accurately predicting class membership for individuals.

^h^Lo-Mendell-Rubin (LMR) p-value from likelihood ratio test for K-1 versus K classes.

**Fig 2:**
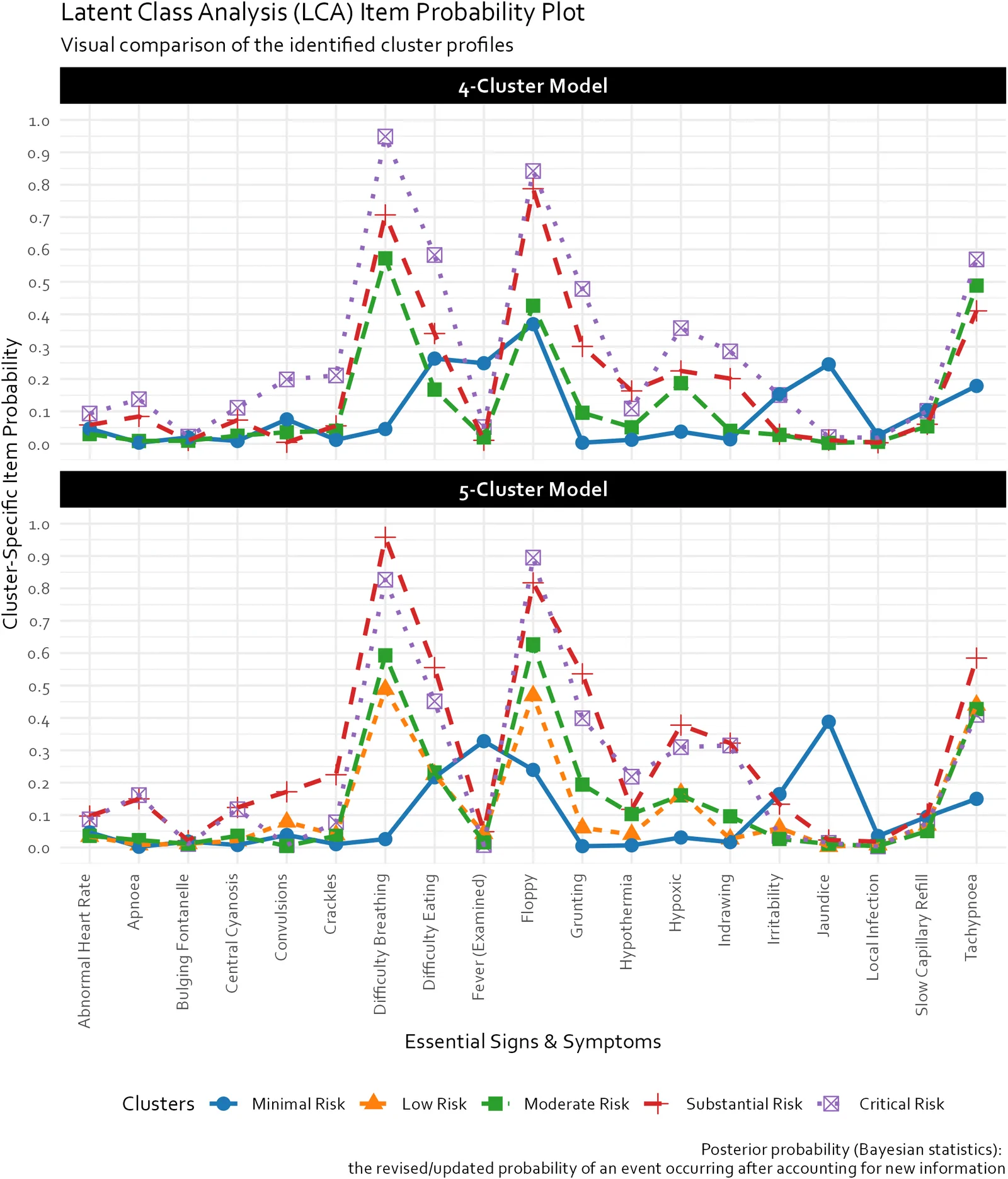
Patterns of signs and symptoms across the different clusters.

Fig 2 illustrates the difference in the probability of pSBI signs and symptoms given cluster membership. The pSBI signs of floppy, tachypnoea, grunting, hypothermia, indrawing, hypoxia, difficulty in feeding and difficulty breathing appear to be useful in distinguishing different clusters (Fig 2). The labels “minimal,” “low,” “moderate,” “substantial,” and “critical” were intended as clinically meaningful descriptors of the relative mortality risk across the clusters; no predefined mortality thresholds were used to assign these labels [37].

There is substantive variation with in-hospital mortality based on cluster membership, with the minimal risk cluster having the least mortality cases (0.98%) and critical risk having the highest mortality cases (67.8%). The same pattern is present in the validation dataset, with 3.06% and 61.76% case fatality rate between the minimal risk cluster and critical risk cluster (Table 3). Patients in the critical risk cluster had low gestational age in weeks and low birth weight while patients in the substantial risk cluster had normal gestational age and birth weight but both clusters had at least 4 pSBI signs/symptoms and substantially high mortality rates of >40% in both the development and validation datasets (Table 3). Admissions in the minimal and low mortality risk clusters had 2 (IQR: 1–3) pSBI signs/symptoms with antibiotics prescribed in ≥45% of the cases within each of these clusters in both the development and validation datasets.

**Table 3:** Characteristics of patients included in model development and external validation.

Indicator	Development (n = 33094)^*a*^					Validation (n = 23704)^*a*^				
	Minimal Risk n = 3368 (10.18%)	Low Risk n = 12907 (39%)	Moderate Risk n = 8879 (26.83%)	Substantial Risk n = 4130 (12.48%)	Critical Risk n = 3810 (11.51%)	Minimal Risk n = 3763 (15.87%)	Low Risk n = 8333 (35.15%)	Moderate Risk n = 5841 (24.64%)	Substantial Risk n = 2940 (12.4%)	Critical Risk n = 2827 (11.93%)
**Neonatal Essential Signs & Symptoms: Binary**										
Jaundice	1510 (44.83%)	21 (0.16%)	94 (1.06%)	99 (2.4%)	53 (1.39%)	1630 (43.32%)	14 (0.17%)	149 (2.55%)	178 (6.05%)	55 (1.95%)
Crackles	29 (0.86%)	469 (3.63%)	304 (3.42%)	993 (24.04%)	298 (7.82%)	51 (1.36%)	394 (4.73%)	154 (2.64%)	751 (25.54%)	199 (7.04%)
Bulging Fontanelle	57 (1.69%)	156 (1.21%)	101 (1.14%)	88 (2.13%)	40 (1.05%)	97 (2.58%)	149 (1.79%)	71 (1.22%)	102 (3.47%)	35 (1.24%)
Local Infection	141 (4.19%)	95 (0.74%)	38 (0.43%)	70 (1.69%)	10 (0.26%)	318 (8.45%)	75 (0.9%)	39 (0.67%)	99 (3.37%)	10 (0.35%)
Apnoea	6 (0.18%)	88 (0.68%)	190 (2.14%)	656 (15.88%)	620 (16.27%)	20 (0.53%)	135 (1.62%)	250 (4.28%)	654 (22.24%)	618 (21.86%)
Difficulty Eating	675 (20.04%)	2892 (22.41%)	1982 (22.32%)	2284 (55.3%)	1667 (43.75%)	925 (24.58%)	1577 (18.92%)	1295 (22.17%)	1837 (62.48%)	1335 (47.22%)
Difficulty Breathing	75 (2.23%)	5997 (46.46%)	5193 (58.49%)	3949 (95.62%)	3095 (81.23%)	172 (4.57%)	3341 (40.09%)	2858 (48.93%)	2762 (93.95%)	2142 (75.77%)
Convulsions	107 (3.18%)	991 (7.68%)	39 (0.44%)	739 (17.89%)	29 (0.76%)	261 (6.94%)	973 (11.68%)	45 (0.77%)	780 (26.53%)	33 (1.17%)
Floppy	656 (19.48%)	5616 (43.51%)	5101 (57.45%)	3183 (77.07%)	3175 (83.33%)	779 (20.7%)	2937 (35.25%)	2984 (51.09%)	2141 (72.82%)	2210 (78.17%)
Indrawing	52 (1.54%)	279 (2.16%)	842 (9.48%)	1427 (34.55%)	1192 (31.29%)	33 (0.88%)	118 (1.42%)	359 (6.15%)	748 (25.44%)	748 (26.46%)
Grunting	15 (0.45%)	675 (5.23%)	1722 (19.39%)	2378 (57.58%)	1514 (39.74%)	67 (1.78%)	408 (4.9%)	715 (12.24%)	1323 (45%)	797 (28.19%)
Slow Capillary Refill	232 (6.89%)	812 (6.29%)	391 (4.4%)	407 (9.85%)	243 (6.38%)	298 (7.92%)	582 (6.98%)	410 (7.02%)	339 (11.53%)	287 (10.15%)
Central Cyanosis	22 (0.65%)	248 (1.92%)	310 (3.49%)	536 (12.98%)	444 (11.65%)	43 (1.14%)	216 (2.59%)	194 (3.32%)	360 (12.24%)	276 (9.76%)
Irritability	592 (17.58%)	774 (6%)	236 (2.66%)	562 (13.61%)	136 (3.57%)	852 (22.64%)	639 (7.67%)	254 (4.35%)	534 (18.16%)	104 (3.68%)
Tachypnoea	358 (10.63%)	4957 (38.41%)	3415 (38.46%)	2077 (50.29%)	1344 (35.28%)	569 (15.12%)	3296 (39.55%)	1968 (33.69%)	1276 (43.4%)	846 (29.93%)
Hypoxic	82 (2.43%)	1883 (14.59%)	1296 (14.6%)	1421 (34.41%)	1033 (27.11%)	149 (3.96%)	1136 (13.63%)	874 (14.96%)	935 (31.8%)	675 (23.88%)
Fever (Examined)	1267 (37.62%)	406 (3.15%)	123 (1.39%)	188 (4.55%)	25 (0.66%)	1605 (42.65%)	452 (5.42%)	215 (3.68%)	370 (12.59%)	45 (1.59%)
Hypothermia	17 (0.5%)	437 (3.39%)	837 (9.43%)	474 (11.48%)	744 (19.53%)	31 (0.82%)	490 (5.88%)	787 (13.47%)	366 (12.45%)	803 (28.4%)
Abnormal Heart Rate	121 (3.59%)	418 (3.24%)	291 (3.28%)	383 (9.27%)	305 (8.01%)	208 (5.53%)	401 (4.81%)	309 (5.29%)	364 (12.38%)	289 (10.22%)
Male	1896 (56.29%)	7814 (60.54%)	4602 (51.83%)	2580 (62.47%)	2043 (53.62%)	2132 (56.66%)	4967 (59.61%)	2890 (49.48%)	1754 (59.66%)	1455 (51.47%)
**Neonatal Essential Signs & Symptoms: Continuous**										
pSBI signs	1 (IQR: 1–2)	2 (IQR: 1–3)	2 (IQR: 1–3)	5 (IQR: 4–6)	4 (IQR: 3–5)	2 (IQR: 1–3)	2 (IQR: 1–3)	2 (IQR: 1–3)	5 (IQR: 4–6)	4 (IQR: 3–5)
Gestational age	38 (IQR: 38–40)	38 (IQR: 38–40)	34 (IQR: 32–35)	38 (IQR: 38–40)	28 (IQR: 27–30)	38 (IQR: 38–40)	39 (IQR: 38–40)	33 (IQR: 32–35)	38 (IQR: 38–40)	28 (IQR: 27–30)
Birth weight	3.1 (IQR: 2.8–3.45)	3.1 (IQR: 2.79–3.435)	1.86 (IQR: 1.6–2.1)	2.99 (IQR: 2.6–3.3)	1.17 (IQR: 0.94–1.4)	3.1 (IQR: 2.78–3.5)	3.1 (IQR: 2.785–3.46)	1.8 (IQR: 1.54–2.08)	3 (IQR: 2.6–3.3525)	1.2 (IQR: 1–1.47)
Age	3 (IQR: 2–4)	1 (IQR: 1−1)	1 (IQR: 1−1)	1 (IQR: 1−1)	1 (IQR: 1−1)	3 (IQR: 2–5)	1 (IQR: 1−1)	1 (IQR: 1−1)	1 (IQR: 1–2)	1 (IQR: 1−1)
Temperature	37.4 (IQR: 36.7–38.4)	36.7 (IQR: 36.3–36.9)	36.5 (IQR: 36.1–36.8)	36.5 (IQR: 36.1–36.8)	36.3 (IQR: 35.675–36.7)	37.8 (IQR: 36.8–38.5)	36.7 (IQR: 36.2–37.1)	36.5 (IQR: 36–36.9)	36.7 (IQR: 36.1–37.3)	36.2 (IQR: 35.2–36.7)
Respiratory Rate	49 (IQR: 45–56)	57 (IQR: 48–66)	57 (IQR: 49–65)	62 (IQR: 50–71)	56 (IQR: 46–65)	49 (IQR: 44–57)	56 (IQR: 48–67)	55 (IQR: 46–64)	60 (IQR: 48–70)	53 (IQR: 43–63)
Heart Rate	138 (IQR: 127–146)	138 (IQR: 127–148)	139 (IQR: 128–149)	140 (IQR: 125–152)	140 (IQR: 124–152)	139 (IQR: 126–150)	138 (IQR: 125–149)	141 (IQR: 127–152)	142 (IQR: 124–156)	140 (IQR: 124–153)
Oxygen saturation	95 (IQR: 94–97)	95 (IQR: 92–97)	95 (IQR: 92–97)	92 (IQR: 84–96)	93 (IQR: 87–96)	95 (IQR: 93–97)	95 (IQR: 92–98)	95 (IQR: 92–98)	92 (IQR: 83–96)	94 (IQR: 87–97)
**Neonatal Essential Interventions**										
Oxygen Prescribed	928 (27.55%)	7035 (54.51%)	5776 (65.05%)	3018 (73.08%)	2907 (76.3%)	398 (10.58%)	3702 (44.43%)	3033 (51.93%)	2093 (71.19%)	1978 (69.97%)
Fluids Prescribed	103 (3.06%)	5109 (39.58%)	4159 (46.84%)	1973 (47.77%)	2038 (53.49%)	72 (1.91%)	1298 (15.58%)	1016 (17.39%)	398 (13.54%)	603 (21.33%)
Feeds Prescribed	627 (18.62%)	1527 (11.83%)	817 (9.2%)	232 (5.62%)	178 (4.67%)	201 (5.34%)	312 (3.74%)	242 (4.14%)	99 (3.37%)	64 (2.26%)
Antibiotics Prescribed	2166 (64.31%)	8641 (66.95%)	6116 (68.88%)	2827 (68.45%)	2672 (70.13%)	1789 (47.54%)	3806 (45.67%)	2362 (40.44%)	1241 (42.21%)	1066 (37.71%)
Phenobarbital Prescribed	105 (3.12%)	1423 (11.03%)	166 (1.87%)	976 (23.63%)	51 (1.34%)	273 (7.25%)	1355 (16.26%)	115 (1.97%)	787 (26.77%)	46 (1.63%)
**Neonatal Outcomes**										
Length Of Stay	5 (IQR: 3–7)	5 (IQR: 3–7)	12 (IQR: 5–23)	4 (IQR: 1–8)	4 (IQR: 1–27)	5 (IQR: 3–7)	5 (IQR: 3–7)	12 (IQR: 5–22)	3 (IQR: 1–8)	5 (IQR: 1–29)
Died	33 (0.98%)	369 (2.86%)	849 (9.56%)	1754 (42.47%)	2583 (67.8%)	115 (3.06%)	355 (4.26%)	524 (8.97%)	1441 (49.01%)	1745 (61.73%)

^*a*^Continuous variable summaries given as median (Interquartile range). Discrete variable summaries given as proportions.

### External validation and transportability of the model-based clustering approach

External validation demonstrated high concordance between cluster membership predicted by the original LCA model and cluster membership derived from an independently fitted LCA model in the external dataset (Table 4). Balanced accuracy ranged from 93.13% to 98.59%, with F1 scores ranging from 91.10% to 97.49% across the five risk classes. Sensitivity was highest for critical (97.53%), moderate (96.96%), and low risk (96.76%) classes, while specificity remained high across all classes (94.90% to 99.66%). Overall, the findings support the reproducibility and generalisability of the five-cluster risk structure, with strong classification performance maintained across all risk classes (Table 4).

**Table 4:** External validation of latent cluster membership using an independently LCA model fitted on the validation dataset.

Metric	Minimal Risk	Low Risk	Moderate Risk	Substantial Risk	Critical Risk
Balanced Accuracy	93.13 (91.46 to 94.77)	95.84 (94.98 to 96.58)	98 (97.29 to 98.66)	94.26 (92.55 to 95.8)	98.59 (97.69 to 99.4)
F1 Score	91.64 (89.52 to 93.57)	93.88 (92.66 to 94.89)	97.01 (96.04 to 97.91)	91.1 (88.81 to 93.29)	97.49 (96.12 to 98.68)
Negative Predictive Value	97.54 (96.89 to 98.17)	98.19 (97.52 to 98.8)	99 (98.55 to 99.43)	98.52 (98.01 to 98.98)	99.66 (99.42 to 99.9)
Positive Predictive Value	97.17 (95.39 to 98.75)	91.15 (89.38 to 92.78)	97.06 (95.77 to 98.31)	92.89 (89.89 to 95.56)	97.45 (95.45 to 98.95)
Recall	86.75 (83.46 to 90.03)	96.76 (95.57 to 97.84)	96.96 (95.61 to 98.24)	89.51 (86.15 to 92.55)	97.53 (95.71 to 99.2)
Sensitivity	86.75 (83.46 to 90.03)	96.76 (95.57 to 97.84)	96.96 (95.61 to 98.24)	89.51 (86.15 to 92.55)	97.53 (95.71 to 99.2)
Specificity	99.54 (99.2 to 99.8)	94.9 (93.85 to 95.86)	99.04 (98.61 to 99.45)	99.04 (98.61 to 99.41)	99.66 (99.38 to 99.86)

The pSBI signs and symptoms used in model-based clustering were 19, excluding birth weight and gestational age and the mortality outcome. From feature selection using xgboost, we subjectively judged 11 (52%) of pSBI signs and symptoms to be sufficiently capable of assigning the patients into the mortality risk clusters using a multivariate logistic model with the outcome as the clusters based on values (Table 5, S4 Fig). From table 5, the cluster membership of any neonate with a pSBI sign/symptom can be determined based on which cluster the patient had the highest probability of being a member of, with the cluster-specific probabilities computed from the sum of the logits based on the presence/absence of the sign or symptom. The transportability assessment demonstrated good overall performance of the simplified classifier in assigning patients to the predefined LCA cluster membership, with balanced accuracy ranging from 83.16% to 95.36% and F1 scores from 78.01% to 93.14% across the five classes. Performance was particularly strong for the low, moderate, and critical Risk classes, while the high specificity across all classes (86.91% to 99.39%) supports the classifier’s ability to reliably distinguish patients belonging to the predefined risk groups (Table 6).

**Table 5:** Parsimonious multivariate logistic model for determining neonatal cluster membership based on pSBI signs and symptoms.

Indicator	Minimal			Low			Moderate			Substantial			Critical		
	Beta	SE	p-value	Beta	SE	p-value	Beta	SE	p-value	Beta	SE	p-value	Beta	SE	p-value
Intercept	4.936	0.146	<0.001	−6.060	0.181	<0.001	−2.202	0.092	<0.001	−7.620	0.165	<0.001	−22.123	0.783	<0.001
Birth weight^*a*^	0.499	0.058	<0.001	1.260	0.035	<0.001	−1.692	0.034	<0.001	0.645	0.050	<0.001	−4.861	0.198	<0.001
Gestational age^*b*^	0.206	0.017	<0.001	0.508	0.010	<0.001	−0.017	0.006	0.004	0.601	0.015	<0.001	−1.807	0.059	<0.001
Early onset age^c^	−5.876	0.134	<0.001	6.592	0.183	<0.001	1.531	0.094	<0.001	−1.861	0.131	<0.001	−0.262	0.370	0.479
Difficulty breathing	−4.215	0.166	<0.001	−0.015	0.040	0.698	−0.045	0.033	0.177	3.578	0.117	<0.001	1.804	0.145	<0.001
Floppy	−2.246	0.082	<0.001	−0.261	0.040	<0.001	−0.218	0.034	<0.001	1.728	0.071	<0.001	2.106	0.160	<0.001
Grunting	−2.633	0.414	<0.001	−2.109	0.059	<0.001	−0.048	0.042	0.256	2.627	0.069	<0.001	1.542	0.135	<0.001
Hypoxic	−1.885	0.174	<0.001	−0.450	0.050	<0.001	−0.508	0.042	<0.001	1.087	0.066	<0.001	1.483	0.144	<0.001
Difficulty feeding	−0.314	0.085	<0.001	−0.675	0.044	<0.001	−0.572	0.037	<0.001	1.295	0.059	<0.001	1.502	0.130	<0.001
Tachypnoea	−1.998	0.092	<0.001	0.161	0.041	<0.001	0.104	0.033	0.002	0.508	0.062	<0.001	−0.176	0.118	0.134
Indrawing	−1.732	0.287	<0.001	−2.145	0.086	<0.001	−0.854	0.053	<0.001	1.941	0.076	<0.001	1.956	0.156	<0.001
Crackles	−2.264	0.323	<0.001	−1.254	0.078	<0.001	−0.571	0.074	<0.001	1.901	0.084	<0.001	1.410	0.233	<0.001
Hypothermia	−2.817	0.374	<0.001	−1.031	0.079	<0.001	−0.649	0.054	<0.001	1.421	0.098	<0.001	1.404	0.152	<0.001
Apnoea	−2.788	0.642	<0.001	−2.653	0.139	<0.001	−1.624	0.090	<0.001	2.006	0.105	<0.001	3.641	0.248	<0.001

^a^Birth weight centred at 2.5 kgs.

^b^Gestational age centred at 37 weeks.

^c^Neonate’s age ≤ 72 hours.

**Table 6:** Performance of the parsimonious multivariate logistic models in predicting cluster membership in the external validation dataset.

Metric	Minimal Risk	Low Risk	Moderate Risk	Substantial Risk	Critical Risk
Balanced Accuracy	83.16 (82.72 to 83.62)	91.87 (91.68 to 92.05)	93.94 (93.71 to 94.19)	85.21 (84.68 to 85.7)	95.36 (95 to 95.71)
F1 Score	78.01 (77.36 to 78.68)	88.22 (87.93 to 88.49)	91.1 (90.78 to 91.42)	78.72 (77.92 to 79.47)	93.14 (92.69 to 93.59)
Negative Predictive Value	94.14 (93.96 to 94.32)	97.94 (97.8 to 98.08)	96.96 (96.81 to 97.13)	96.32 (96.17 to 96.47)	98.9 (98.81 to 98.99)
Positive Predictive Value	92.72 (92.2 to 93.26)	81.02 (80.58 to 81.45)	91.65 (91.21 to 92.03)	87.05 (86.15 to 87.98)	95.02 (94.5 to 95.59)
Recall	67.33 (66.43 to 68.24)	96.83 (96.61 to 97.04)	90.55 (90.08 to 91.03)	71.86 (70.77 to 72.85)	91.32 (90.62 to 92.03)
Sensitivity	67.33 (66.43 to 68.24)	96.83 (96.61 to 97.04)	90.55 (90.08 to 91.03)	71.86 (70.77 to 72.85)	91.32 (90.62 to 92.03)
Specificity	99 (98.93 to 99.08)	86.91 (86.61 to 87.23)	97.33 (97.19 to 97.47)	98.57 (98.47 to 98.67)	99.39 (99.33 to 99.46)

**Note**: The 95% confidence interval was determined through computing the metrics in each of the 1000 iterations of sampling with replacement of 10% of the external validation dataset

### Mortality risk prediction performance based on clusters

**Fig 3:**
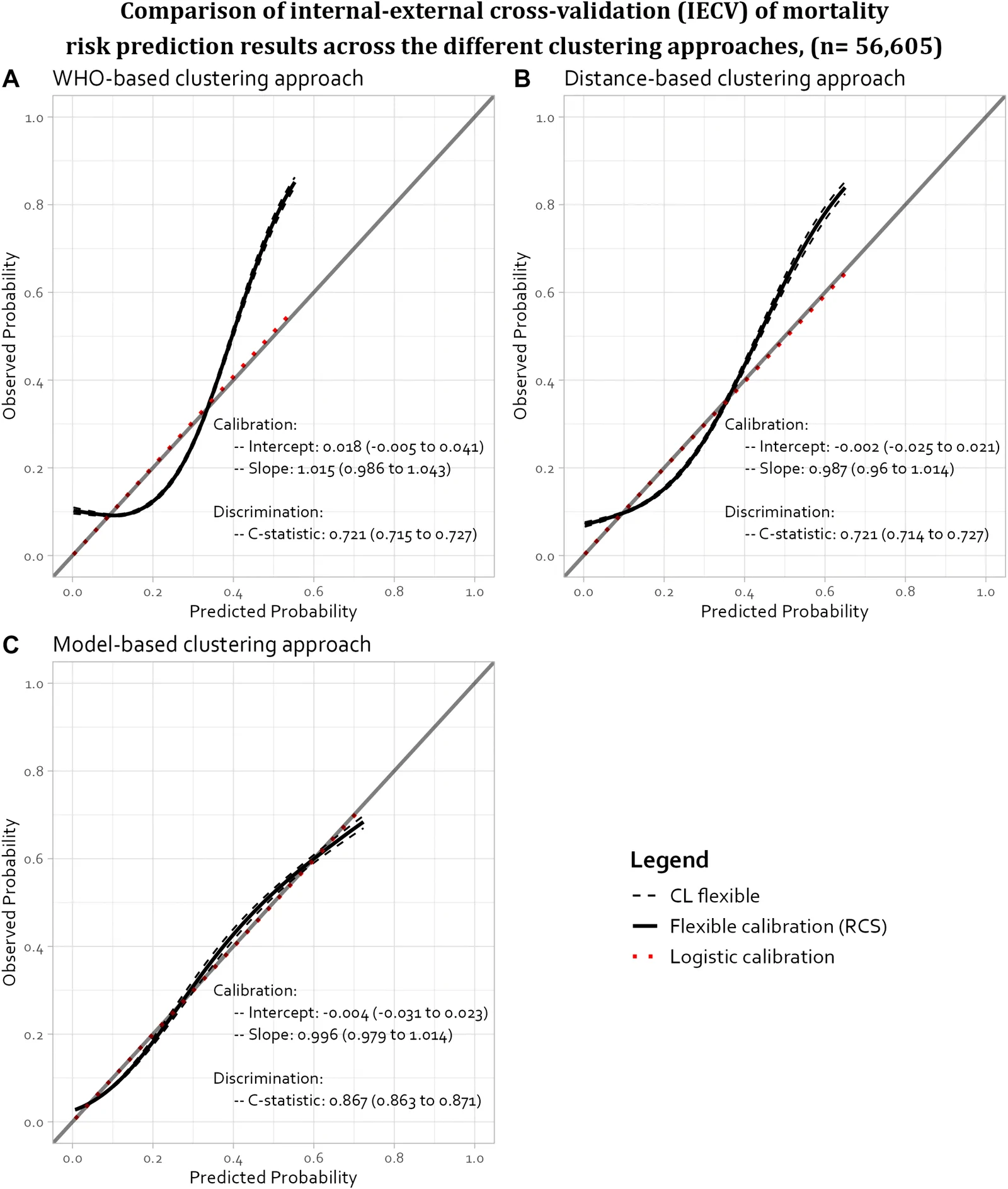
Comparison of clustering approach’s mortality risk predictive accuracy.

Fig 3 compares the performance of the three clustering approaches in predicting mortality risk. Overall, 33004/33094 (99%) admissions of the development dataset and 23601/23704 (99%) of the validation dataset were eligible for this comparison; The omitted admissions were excluded since their clinical signs and symptoms did not align with any of the predefined WHO clusters (S1 Table), due to variable missingness, posing a challenge in assigning them to a WHO cluster.

The c-statistic (discrimination) for the WHO expert-based clustering approach was 0.721 (95% CI: 0.715 to 0.727) with a calibration intercept of 0.018 (95% CI: −0.005 to 0.041) and calibration slope of 1.015 (95% CI: 0.986 to 1.043). The c-statistic (discrimination) for the distance-based clustering approach was 0.721 (95% CI: 0.714 to 0.727) while its calibration intercept was −0.002 (95% CI: −0.025 to 0.021) and calibration slope was 0.987 (95% CI: 0.96 to 1.014). The WHO and distance-based clustering approach calibration curves both showed substantive variances across the mortality risk spectrum with over-estimation of mortality at the lowest predicted risks and under-estimation at moderate to high risks of mortality (Fig 3).

The model-based clustering approach had the highest c-statistic (discrimination) of 0.867 (95% CI: 0.863 to 0.871), with a calibration intercept of −0.004 (95% CI: −0.031 to 0.023) and a calibration slope of 0.996 (95% CI: 0.979 to 1.043). The model-based clustering approach’s calibration curve showed close and relatively better alignment between the predicted risks and observed outcomes across the probability range compared to the alternative clustering approaches (Fig 3).

## Discussion

### Summary of findings

This study used a pragmatic approach with a limited set of signs easy to use by clinicians (usually busy junior clinicians) to identify residual heterogeneity in the neonates admitted with signs indicative of sepsis, that can be used to inform antibiotic use treatment decisions and strategies. From applying model-based clustering approaches on routinely collected neonatal data at hospital admission, we identified five clinically distinct clusters of neonates with pSBI managed for sepsis, labelled based on the mortality risk in each cluster: minimal risk, low risk, moderate risk, substantial risk and critical risk (Table 3, Fig 2). From external validation, we further demonstrate that the model-based clusters outperform distance-based and WHO expert-based clustering approaches in predicting in-hospital mortality based on discrimination and calibration statistics (Fig 3). Among the 19 pSBI signs/symptoms collected at admission and used in the initial model-based clustering, a subset of 11 pSBI signs and symptoms together with birth weight, gestational age, early-onset age clinical variables were sufficient to determine cluster membership of a patient and subsequently the associated mortality risk from pSBI at admission (Table 5, Table 6).

Hypothermia, indrawing, floppy, tachypnoea, grunting, hypoxia, difficulty in feeding and difficulty breathing were key in distinguishing the clusters. These signs were mostly prevalent in the substantial and critical risk groups with tachypnoea being more common in the substantial risk cluster while hypothermia and indrawing were more common in the critical risk cluster. The critical risk cluster also comprised of neonates with low gestational age and birthweight while those in substantial risk cluster had normal gestation age and birthweight yet still faced substantially high mortality rate. The moderate risk cluster on the other hand, included neonates with low birth weight but demonstrated a relatively lower rate of mortality risk, highlighting the heterogeneity in mortality risk beyond birth-related factors.

### Comparison to other findings

The findings of this study contribute to the growing evidence of how unsupervised machine learning methods might identify meaningful phenotypes in neonatal sepsis populations. For instance, Seymour et al. and Jang et al. [4,15] employed k-means clustering and identified up to four clusters. Sinha et al. used model-based clustering and derived two clusters, unlike our findings. These studies used patients with different characteristics and clinical variables whose availability at the time of admission varied considerably. Unlike this current study which used routinely collected clinical data from low-resource neonatal units in an SSA country and relied only on clinical signs and symptoms, some of the other prior studies were conducted in high-income settings and included a set of variables combining both clinical and laboratory parameters that are typically unavailable at neonatal admission such as white blood cell count, platelet count, C-reactive protein, premature neutrophil count and serum levels.

Furthermore, these studies neither directly assessed the external validity of the clustering approach to classify new patients into the identified clusters nor did they provide the probability of patients having a combination of signs and symptoms given their cluster membership. These methodological differences highlight how selection of variables and validation approaches influence findings across studies. The observed differences in mortality risk among the clusters in this study and how the risk differs based on the probability of pSBI in each cluster agree with findings from Puri et al [56] although our findings support five distinct neonatal sepsis clusters. Hypothermia, among the signs previously identified as strongly predictive of death, was particularly common in the critical risk cluster of our study which had the highest mortality risk.

### Implications of findings

The clusters identified through model-based unsupervised learning approach reliably stratify neonatal sepsis into patient sub-populations predictive of in-hospital mortality at admission, outperforming the WHO expert-based and distance-based clustering methods as exemplified by the reported discrimination and calibration statistics (Fig 3). This work shows that there is residual heterogeneity in the neonatal sepsis in-hospital admissions linked to mortality as an outcome. However, mortality may also have been influenced by differences in treatment and other aspects of care received during hospitalisation, which were not accounted for in the clustering model. Therefore, the observed differences in mortality between clusters should not be interpreted as reflecting differences in underlying disease severity alone, and prospective studies are needed to disentangle the contribution of baseline phenotype from subsequent treatment and care. The improved mortality risk stratification exemplified by our model-based clustering approach provides a foundation for more tailored clinical decision making and potential development of phenotype-specific treatment guidelines that does not neglect the typical multimorbid nature of neonatal sepsis admissions in routine SSA hospitals. Additionally, the identification of substantial and critical mortality risk phenotype with distinct clinical profiles could guide prioritisation of care or more aggressive interventions, potentially improving outcomes in these groups. In the context of the growing global threat of antimicrobial resistance (AMR), the finding that more than 45% of neonates admitted to CIN facilities in the minimal and low mortality-risk clusters received antibiotics warrants further evaluation of whether antibiotic use could be safely reduced in some of these patients. The clusters identified in this study provide a more granular stratification than the WHO categories and could provide a basis for prospectively evaluating whether different antibiotic treatment strategies, such as shorter intravenous courses or, where clinically appropriate, oral therapy, can be used safely and effectively.

These findings can either (a) be used to augment the clinical judgement of clinicians in risk stratification of neonatal sepsis patients at admission [57] and/or (b) inform the design and implementation of emulated trials [58] to evaluate different antibiotic treatment strategies for neonatal sepsis at admission informed by the identified clusters [59,60].

By relying on clinical signs and symptoms readily available at admission, the model-based clusters have better chances of being incorporated into routine clinical workflows at the point of care in similar SSA hospital settings [18,25], with the potential to enhance early tailored interventions and treatment to ensure critical care is efficiently administered where it is most needed.

### Strengths and limitations of findings

This study has several notable strengths. First, it is based on a large multi-centre cohort of neonatal admission (n = 56798) across 21 hospitals over three years, ensuring geographical and time coverage thus increasing confidence in the applicability of the modelling approaches across diverse neonatal care contexts. The inclusion criteria ensured the study sample was reflective of the typical multi-morbidity of neonatal admissions, thereby strengthening the real-world relevance of the findings. From a methodological standpoint, this study advances neonatal sepsis phenotyping by moving beyond conventional distance-based clustering techniques to more superior probabilistic, multivariate model-based approaches, which unlike distance-based methods, do not use a forced-choice mechanism to assign patients to clusters.

The absence of microbiological validation to confirm the presence of bacteria indicative of sepsis is a limitation to this study. Since the aim was to classify neonates based on clinical pSBI signs in recognition of limited laboratory capacity in similar SSA hospital settings [18,25], the clusters should hence be interpreted as clinical phenotypes rather than pathogen confirmed syndromes. Future work would involve validating and extending this study’s findings by integrating microbiological testing with clinical clustering to ascertain whether the data-driven phenotypic clusters truly reflect bacterial infections. Prospective studies could also evaluate whether these groups respond differently to interventions or management strategies, providing insights into their clinical utility and potential to inform targeted risk-based neonatal care.

## Conclusions

In this retrospective cohort study, we used pSBI signs that are readily available at neonatal admission across many SSA hospital settings for model-based clustering approach and identified five clinically distinct clusters differentiated by mortality risk: minimal, moderate, substantial and critical risk. From discrimination and calibration statistics, the model-based clustering approach outperformed clustering patients using WHO expert-based guidelines and typical distance-based clustering approaches. These clusters demonstrate the potential to complement clinical judgement in mortality risk stratification at admission and could inform future randomised clinical trials to test whether triage guided by these clusters could improve clinical outcomes such as mortality and length of hospital stay. Future work could also integrate microbiological validation to confirm whether clusters map onto pathogen-specific syndromes and investigate possible variation in treatment responses across the clusters. These efforts could help strengthen the case for more targeted and risk-based neonatal sepsis management, informed by data-driven clustering.

### Consent for publication

This study is published with the permission of the Director of Kenya Medical Research Institute (KEMRI).

## Supporting information

S1 Table WHO guidance on the management of neonates based on the possible Signs of Bacterial Infections (pSBI). WHO guidance spans newborns aged 0–59 days but this analysis is restricted to those aged 0–28 days (i.e., neonatal period).
(DOCX)

S2 Table Latent class analysis model performance metrics. (DOCX)

S3 Table Measures for model’s performance assessment. (DOCX)

S4 Table Case fatality rate by hospital. Data from the included patients. Numerator: total number of deaths; Denominator: total number of admissions to the NBU.
(DOCX)

S1 Fig pSBI signs/symptoms in the newborn patients included. pSBI signs and symptoms grouped by the different datasets.
(TIF)

S2 Fig Missingness in the pSBI signs/symptoms in the newborn patients included in analyses. Patterns of pSBI signs and symptoms missingness grouped by the different datasets.
(TIF)

S3 Fig Top 4 common diagnoses for neonates with ≥1 pSBI signs/symptoms. Common admission diagnoses for neonates managed for sepsis (n = 8012), excluding sepsis diagnosis.
(TIF)

S4 Fig Feature importance for determining cluster membership, derived from use of extreme gradient boosted regression trees (xgboost). (TIF)

S1 Text Feature selection using extreme gradient boosting. (DOCX)

## Abbreviations

CIN: clinical information network
EHRs: electronic health records
HICs: high income countries
HCWs: health care workers
KPA: Kenya paediatric association
LCA: latent class analysis
LMICs: low and middle-income countries
MoH: ministry of health
NBU: newborn unit
pSBI: possible serious bacterial infection (pSBI)
SERU: KEMRI’s scientific and ethics review unit
SSA: sub-Saharan Africa
WHO: World Health Organization
