# Supplementary material for: A cluster analysis of neonates using clinical signs of possible serious bacterial infection at hospital admission in Kenya: A retrospective multicentre cohort study
Source: PLoS One. 2026 Sep 24;21(9):e0359212. doi: 10.1371/journal.pone.0359212 (PMC13606953; doi:10.1371/journal.pone.0359212)
Supplement: Supplementary file 1 [file pone.0359212.s001.docx]

**S1 Table: WHO guidance on the management of neonates based on the possible Signs of Bacterial Infections (pSBI)**

| **WHO Classification [14]** | **Characteristics** | **Antibiotic Recommendation** |
| --- | --- | --- |
| Fast breathing (Respiratory rate ≥ 60 breaths/min) as the only sign of illness | 0-6 days old | Oral amoxicillin (50 mg/kg per dose, twice daily) for 7 days. |
|  | 7-59 days old | Oral amoxicillin (50 mg/kg per dose, twice daily) for 7 days. |
| Clinical Severe Infection: at least one sign of severe infection e.g. movement only when stimulated, not feeding well, fever (≥ 38°C) or hypothermia (< 35.5°C) and severe chest in-drawing) | 0-59 days old | IM Gentamicin (5–7.5 mg/kg once daily for 7 days) + Oral Amoxicillin (50 mg/kg per dose, twice daily for 7 days)  Alternative: IM Gentamicin (5–7.5 mg/kg once daily for 2 days) + Oral Amoxicillin (50 mg/kg per dose, twice daily for 7 days) |
| Critical Illness: Presence of any of the following signs: convulsions, unable to feed at all, no movement on stimulation, unable to cry, bulging fontanelle, cyanosis. | 0-59 days | IM Ampicillin (twice daily) + IM Gentamicin (5–7.5 mg/kg once daily)  Alternative: IM Ceftriaxone (once daily) ± IM Gentamicin (once daily)  Alternative: IM Benzyl Penicillin (twice daily) + IM Gentamicin (once daily)  Alternative: IM Procaine Penicillin (once daily) + IM Gentamicin (once daily) |

Note: World Health Organization. (2015) “GUIDELINE: Managing possible serious bacterial infection in young infants when referral is not feasible.”
