# Supplementary material for: A cluster analysis of neonates using clinical signs of possible serious bacterial infection at hospital admission in Kenya: A retrospective multicentre cohort study
Source: PLoS One. 2026 Sep 24;21(9):e0359212. doi: 10.1371/journal.pone.0359212 (PMC13606953; doi:10.1371/journal.pone.0359212)
Supplement: Supplementary file 2 [file pone.0359212.s002.docx]

**S2 Table: Latent class analysis model performance metrics**

|  | Metric | Definition |
| --- | --- | --- |
| 1. | Bayesian Information Criterion (BIC) | The BIC rewards parsimony in models and can be used to compare competing LCA solutions. Lower BICs indicate better fit [1, 2]. |
| 2. | Vuong-Lo-Mendell-Rubin adjusted likelihood ratio test | Provides a p-value, which indicates if one model is statistically better than another (e.g. model with K classes is better than one with K-1 classes) [1, 2]. |
| 3. | The average latent class posterior probability (ALCPP) | The average probability of the class model accurately predicting class membership for individuals. Probabilities between .80 and .90 are acceptable. |
| 4. | Entropy | It indicates how accurately the model defines classes. In general, an entropy value close to 1 is ideal and above .8 is acceptable [3, 4]. |
| 5. | Number of sample members in each class | There are no existing guidelines on determining class size. While consensus is that classes should not contain less than 5% of the sample, the important issue is whether the small class makes conceptual sense [5]. |

1.	Lo, Y., Nancy R. Mendell, and D.B. Rubin, *Testing the number of components in a normal mixture.* Biometrika, 2001. **88**(3): p. 767-778.

2.	Nylund-Gibson, K. and A.Y. Choi, *Ten frequently asked questions about latent class analysis.* Translational issues in psychological science, 2018. **4**(4): p. 440-461.

3.	Celeux, G., G. Soromenho, and *An entropy criterion for assessing the number of clusters in a mixture model.* 1996. **13**: p. 195-212.

4.	Wang, M.C., et al., *Performance of the entropy as an index of classification accuracy in latent profile analysis: A Monte Carlo simulation study.* Acta Psychologica Sinica, 2017. **49**(11): p. 1473-1482.

5.	Weller, B.E., N.K. Bowen, and S.J. Faubert, *Latent class analysis: a guide to best practice.* Journal of black psychology, 2020. **46**(4): p. 287-311.
