# Supplementary material for: A cluster analysis of neonates using clinical signs of possible serious bacterial infection at hospital admission in Kenya: A retrospective multicentre cohort study
Source: PLoS One. 2026 Sep 24;21(9):e0359212. doi: 10.1371/journal.pone.0359212 (PMC13606953; doi:10.1371/journal.pone.0359212)
Supplement: Supplementary file 3 [file pone.0359212.s003.docx]

**S3 Table: Measures for model’s performance assessment**

1. Calibration (adapted from Riley et al [1])

This is how close the predicted mortality event is close to the observed mortality event. This measure has two key components:

(a) Calibration slope

The calibration slope measures the agreement between the observed and predicted risks of the event (outcome) across the whole range of predicted values. For a perfectly calibrated model, we expect to see that, in 100 individuals with a predicted risk of *r*% from our model, *r* of the 100 truly have the outcome of interest (i.e. death in this case). The slope should ideally be 1. A slope < 1 indicates that some predictions are too extreme (e.g. predictions close to 1 are too high, and predictions close to 0 are too low), and a slope > 1 indicates predictions are too narrow. A calibration slope < 1 is often observed in validation studies, consistent with over-fitting in the original model development

(b) Calibration-in-the-large (calibration intercept)

The calibration intercept compares the mean of all predicted risks with the mean observed risk, i.e. on average how close is predicted to observed in the whole dataset. This parameter hence indicates the extent that predictions are systematically too low or too high. It can be well assessed graphically, in a plot with predictions on the *x*-axis and the observed endpoint on the *y*-axis. The observed values on the *y*-axis are 0 or 1 (e.g. dead/alive), while the predictions on the *x*-axis range between 0 and 100% with the intercept representing calibration-in-the-large

2. Discrimination (adapted from Riley et al [1])

This is a measure of a prediction model’s separation between those with or without the outcome, usually represented by the c-statistic, which is also known as the concordance index or, for binary outcomes, the area under the receiver operating characteristic (AUROC) curve. It gives the probability that for any randomly selected pair of individuals, one who died and one who survived (outcome), the model assigns a higher probability to the individual who died (outcome). A value of 1 indicates the model has perfect discrimination, while a value of 0.5 indicates the model discriminates no better than chance

1.	Riley, R.D., et al., *External validation of clinical prediction models using big datasets from e-health records or IPD meta-analysis: Opportunities and challenges.* BMJ, 2019. **365**.
