# Supplementary material for: A cluster analysis of neonates using clinical signs of possible serious bacterial infection at hospital admission in Kenya: A retrospective multicentre cohort study
Source: PLoS One. 2026 Sep 24;21(9):e0359212. doi: 10.1371/journal.pone.0359212 (PMC13606953; doi:10.1371/journal.pone.0359212)
Supplement: Supplementary file 4 [file pone.0359212.s004.docx]

**S4 Table. Case fatality rate by hospital**

| **Dataset type** | **Hospital** | **Mortality Rate^a^** |
| --- | --- | --- |
| Development | H12 | 347/3221 (10.77%) |
| Development | H13 | 962/5484 (17.54%) |
| Development | H14 | 800/5020 (15.94%) |
| Development | H16 | 428/2703 (15.83%) |
| Development | H17 | 988/5440 (18.16%) |
| Development | H19 | 756/4456 (16.97%) |
| Development | H7 | 872/4047 (21.55%) |
| Development | H9 | 435/2723 (15.98%) |
| Validation | H1 | 672/3284 (20.46%) |
| Validation | H10 | 104/1135 (9.16%) |
| Validation | H11 | 530/2346 (22.59%) |
| Validation | H15 | 192/879 (21.84%) |
| Validation | H18 | 171/1566 (10.92%) |
| Validation | H2 | 121/795 (15.22%) |
| Validation | H20 | 497/3084 (16.12%) |
| Validation | H21 | 144/1089 (13.22%) |
| Validation | H3 | 229/2152 (10.64%) |
| Validation | H4 | 315/1289 (24.44%) |
| Validation | H5 | 530/2930 (18.09%) |
| Validation | H6 | 580/2389 (24.28%) |
| Validation | H8 | 95/766 (12.4%) |

^a^Data from the included patients**.** Numerator: total number of deaths; Denominator: total number of admissions to the NBU
