# Supplementary material for: A cluster analysis of neonates using clinical signs of possible serious bacterial infection at hospital admission in Kenya: A retrospective multicentre cohort study
Source: PLoS One. 2026 Sep 24;21(9):e0359212. doi: 10.1371/journal.pone.0359212 (PMC13606953; doi:10.1371/journal.pone.0359212)
Supplement: Supplementary file 5 [file pone.0359212.s009.docx]

**S1 Text: Feature selection using extreme gradient boosting**

Feature selection was performed using a supervised gradient-boosting approach based on extreme gradient boosting (XGBoost). All categorical predictors were explicitly encoded as categorical variables and supplied directly to the XGBoost model using its native categorical feature handling. Rather than applying a separate univariate screening procedure, candidate predictors were retained for model development and their contribution to predictive performance was assessed within the multivariable modelling framework.

An XGBoost multiclass classifier was fitted to predict the five-category outcome using a learning rate of 0.05 and up to 1,000 boosting iterations. Model complexity and regularisation parameters were optimised using an exhaustive grid search. The parameters explored included maximum tree depth (2 to 4), feature subsampling (colsample_bytree: 0.60 and 0.75), observation subsampling (subsample: 0.60 and 0.75), minimum loss reduction required for further splitting (gamma: 0.1, 1, and 5), L1 regularisation (reg_alpha: 0.01, 0.05 and 0.10), and minimum child weight (1 to 8).

To account for the clustered nature of the data and avoid information leakage between hospitals, model selection used Stratified Group K-fold cross-validation, with the number of folds equal to the number of participating hospitals. Hospital was specified as the grouping variable, such that observations from the same hospital were not distributed across both training and validation folds. Stratification was used simultaneously to preserve the distribution of the five outcome classes across folds. Hyperparameter combinations were evaluated using the macro-averaged F1 score, which gives equal weight to each outcome class and is therefore appropriate where class frequencies may be imbalanced.

The hyperparameter combination achieving the highest mean cross-validated macro-F1 score was selected as the final model specification. This procedure therefore provided a data-driven approach to identifying the optimal model complexity and regularisation while accounting for both outcome imbalance and hospital-level clustering. The resulting model was subsequently used for the downstream assessment of predictor importance and model performance.

To improve interpretability of the final XGBoost model, SHapley Additive exPlanations (SHAP) values were used to quantify the contribution of individual predictors to each model prediction. For each observation and outcome class, the SHAP value represents the change in the model's output attributable to a given predictor relative to the model's expected output. Thus, a positive SHAP value indicates that the predictor contributed towards a higher model output for the corresponding class, whereas a negative value indicates a contribution towards a lower model output. The magnitude of the SHAP value reflects the strength of the predictor's contribution to that prediction, with larger absolute values indicating greater influence.

Because the data were clustered by hospital, hospital was used as the grouping variable in the cross-validation procedure. Specifically, hospital assignment was used to define the cross-validation folds but was not treated as a predictive feature. Consequently, hospital itself does not have a SHAP value and should not be interpreted as a predictor of the outcome. Instead, the use of hospital as a grouping variable ensures that observations from the same hospital were not simultaneously used for model training and validation, thereby reducing the potential for hospital-specific patterns or correlated observations to inflate predictive performance.

The SHAP analysis should therefore be interpreted as identifying patient-level predictors that drive model predictions within a model evaluated across hospital-level clusters, rather than identifying predictors of hospital membership. For example, if a clinical feature has a positive SHAP value for a particular outcome class, this indicates that the observed value of that feature moved the model prediction towards that class for that patient, irrespective of the hospital in which the patient was observed. Conversely, a negative SHAP value indicates that the feature moved the prediction away from that class.

For overall interpretation, the mean absolute SHAP value across observations was used to quantify the relative importance of predictors. Higher mean absolute SHAP values indicate predictors that, on average, exerted a greater influence on model predictions. Where SHAP values were examined separately by hospital, differences in their distributions were interpreted as variation in the model's use of predictors across hospital clusters, rather than evidence that hospital membership itself caused the observed differences. Such hospital-specific SHAP patterns may indicate differences in case mix, clinical practice, measurement patterns, or the relationship between predictors and outcomes across settings, but they should not be interpreted causally.

The combination of grouped cross-validation and SHAP interpretation therefore distinguishes two related but conceptually different components of the analysis: hospital clustering was incorporated during model evaluation to assess generalisability across facilities, while SHAP values were used to explain which patient-level features contributed to the model's predictions. This approach reduces the risk of attributing hospital-specific variation to individual predictors while allowing potential heterogeneity in predictor contributions across hospital settings to be explored.
