# Supplementary material for: A cluster analysis of neonates using clinical signs of possible serious bacterial infection at hospital admission in Kenya: A retrospective multicentre cohort study
Source: PLoS One. 2026 Sep 24;21(9):e0359212. doi: 10.1371/journal.pone.0359212 (PMC13606953; doi:10.1371/journal.pone.0359212)
Supplement: Supplementary file 6 [file pone.0359212.s010.docx]

PONE-D-25-67644: A cluster analysis of neonates using clinical signs of possible serious bacterial infection at hospital admission in Kenya: A retrospective multicentre cohort study.

PLOS One

Dear Dr. Tuti,

Thank you for submitting your manuscript to PLOS ONE. After careful consideration, we feel that it has merit but does not fully meet PLOS ONE’s publication criteria as it currently stands. Therefore, we invite you to submit a revised version of the manuscript that addresses the points raised during the review process.

Please submit your revised manuscript by Oct 01 2026 11:59PM. If you will need more time than this to complete your revisions, please reply to this message or contact the journal office at [plosone@plos.org](mailto:plosone@plos.org). When you're ready to submit your revision, log on to <https://www.editorialmanager.com/pone/> and select the 'Submissions Needing Revision' folder to locate your manuscript file.

Please include the following items when submitting your revised manuscript:

- A letter that responds to each point raised by the academic editor and reviewer(s). You should upload this letter as a separate file labeled 'Response to Reviewers'.
- A marked-up copy of your manuscript that highlights changes made to the original version. You should upload this as a separate file labeled 'Revised Manuscript with Track Changes'.
- An unmarked version of your revised paper without tracked changes. You should upload this as a separate file labeled 'Manuscript'.

If you would like to make changes to your financial disclosure, please include your updated statement in your cover letter. Guidelines for resubmitting your figure files are available below the reviewer comments at the end of this letter.

If applicable, we recommend that you deposit your laboratory protocols in protocols.io to enhance the reproducibility of your results. Protocols.io assigns your protocol its own identifier (DOI) so that it can be cited independently in the future. For instructions see: [https://journals.plos.org/plosone/s/submission-guidelines#loc-laboratory-protocols](https://eur01.safelinks.protection.outlook.com/?url=https%3A%2F%2Ftrack.editorialmanager.com%2FCL0%2Fhttps%3A%252F%252Fjournals.plos.org%252Fplosone%252Fs%252Fsubmission-guidelines%2523loc-laboratory-protocols%2F1%2F010f01a00ee05503-10ee4a0c-827a-4d11-af07-7aa3a9f42b5b-000000%2F3PqR0egGWAa5Sc5VbwVlp1K0Pmh7datd5YBa2tFCINw%3D258&data=05%7C02%7Cttuti%40kemri-wellcome.org%7Cfc003f2a4d304c6f670708defc3b1394%7Ca5c0a820c8874727ac66403237d8c389%7C0%7C0%7C639225529192645547%7CUnknown%7CTWFpbGZsb3d8eyJFbXB0eU1hcGkiOnRydWUsIlYiOiIwLjAuMDAwMCIsIlAiOiJXaW4zMiIsIkFOIjoiTWFpbCIsIldUIjoyfQ%3D%3D%7C0%7C%7C%7C&sdata=xL3wl84p3%2F6WwHrfbHeugaKTmVQsJUw%2F%2BCFk470iyFs%3D&reserved=0). Additionally, PLOS ONE offers an option for publishing peer-reviewed Lab Protocol articles, which describe protocols hosted on protocols.io. Read more information on sharing protocols at [https://plos.org/protocols?utm_medium=editorial-email&utm_source=authorletters&utm_campaign=protocols](https://eur01.safelinks.protection.outlook.com/?url=https%3A%2F%2Ftrack.editorialmanager.com%2FCL0%2Fhttps%3A%252F%252Fplos.org%252Fprotocols%253Futm_medium%3Deditorial-email%2526utm_source%3Dauthorletters%2526utm_campaign%3Dprotocols%2F1%2F010f01a00ee05503-10ee4a0c-827a-4d11-af07-7aa3a9f42b5b-000000%2F0vmtJgEfuATGx641VWn24q2jHuIXXC96caaMmBrcy10%3D258&data=05%7C02%7Cttuti%40kemri-wellcome.org%7Cfc003f2a4d304c6f670708defc3b1394%7Ca5c0a820c8874727ac66403237d8c389%7C0%7C0%7C639225529192712666%7CUnknown%7CTWFpbGZsb3d8eyJFbXB0eU1hcGkiOnRydWUsIlYiOiIwLjAuMDAwMCIsIlAiOiJXaW4zMiIsIkFOIjoiTWFpbCIsIldUIjoyfQ%3D%3D%7C0%7C%7C%7C&sdata=HUDwD8dVKaxrNIkzFY2E5VT%2BV9tke%2BJJj8%2F5lQA5tCY%3D&reserved=0).

As the corresponding author, your ORCID iD is verified in the submission system and will appear in the published article. PLOS supports the use of ORCID, and we encourage all coauthors to register for an ORCID iD and use it as well. Please encourage your coauthors to verify their ORCID iD within the submission system before final acceptance, as unverified ORCID iDs will not appear in the published article. *Only* the individual author can complete the verification step; PLOS staff *cannot* verify ORCID iDs on behalf of authors.

We look forward to receiving your revised manuscript.

Kind regards,

Khin Thet Wai, MBBS, MPH, MA
Academic Editor
PLOS One

Dear Dr. Wai,

Thank you for the opportunity to revise and resubmit our manuscript, PONE-D-25-67644: “*A cluster analysis of neonates using clinical signs of possible serious bacterial infection at hospital admission in Kenya: A retrospective multicentre cohort study*.**”**

We are grateful for your careful consideration of our manuscript and for the constructive feedback provided during the review process. We have carefully considered the comments from the Academic Editor and reviewers and have revised the manuscript accordingly.

In the revised manuscript, we have addressed each of the points raised and have made changes to improve the clarity, methodological description, interpretation, and presentation of the findings. We have provided a detailed, point-by-point response to the academic editor and reviewers in the accompanying Response to Reviewers document, with specific references to the corresponding changes in the revised manuscript.

In accordance with the journal's requirements, we have also submitted:

1. Response to Reviewers: Providing a detailed response to each comment raised by the Academic Editor and reviewers.
2. Revised Manuscript with Track Changes: Highlighting all changes made in response to the review.
3. Manuscript: The clean, unmarked version of the revised manuscript.

We have also reviewed the manuscript and accompanying submission materials in relation to the journal's requirements, including the financial disclosure and author information.

We appreciate the opportunity to revise our manuscript and thank you and the reviewers for the time and consideration given to our work. We hope that the revised manuscript satisfactorily addresses the concerns raised and is now suitable for publication in *PLOS ONE*.

Kind regards,

**Timothy Tuti**
Corresponding Author

Journal Requirements:

When submitting your revision, we need you to address these additional requirements.

1. Please ensure that your manuscript meets PLOS ONE's style requirements, including those for file naming. The PLOS ONE style templates can be found at

<https://journals.plos.org/plosone/s/file?id=wjVg/PLOSOne_formatting_sample_main_body.pdf> and 
<https://journals.plos.org/plosone/s/file?id=ba62/PLOSOne_formatting_sample_title_authors_affiliations.pdf>

We thank the Academic Editor for this guidance. In response, we have carefully reviewed and updated the manuscript to align with the PLOS ONE formatting and style requirements. Specifically, we have revised the formatting of the title page and author affiliations (lines 1 to 21), the main body of the manuscript, including subheadings (e.g., lines 26, 57, 129 to 130, 145, 292, 421, and 520), as well as the table formatting and in-text referencing. We have also reviewed the presentation of references within the main text, with examples of the revised formatting provided in lines 294 to 353 and 851 to 856. In addition, we have reviewed the manuscript file naming and submission files in accordance with the journal's requirements.

We believe that the revised manuscript and accompanying files now conform to the PLOS ONE style and formatting requirements illustrated in the provided templates.

2. We note that the grant information you provided in the ‘Funding Information’ and ‘Financial Disclosure’ sections do not match.  When you resubmit, please ensure that you provide the correct grant numbers for the awards you received for your study in the ‘Funding Information’ section.

In response to the editor’s comment, we have updated the funding information provided in the submission system to make sure it includes the missing information (“*Additional support was provided by a Wellcome Trust core grant awarded to the KEMRI-Wellcome Trust Research Programme (#092654)*.”) We have also removed the information in manuscript’s declarations section as requested by the editor during resubmission.

3. Thank you for stating the following financial disclosure:  “Wellcome Trust Early Career Fellowship (#227562/Z/23/Z) awarded to TT and a Wellcome Trust Senior Fellowship (#097170) awarded to ME”. Please state what role the funders took in the study. If the funders had no role, please state: "The funders had no role in study design, data collection and analysis, decision to publish, or preparation of the manuscript." If this statement is not correct you must amend it as needed. Please include this amended Role of Funder statement in your cover letter; we will change the online submission form on your behalf.

In response to the Editor’s comment, we have updated the funding information in lines 566 to 567 of the revised manuscript to explicitly state that *“The funders had no role in study design, data collection and analysis, decision to publish, or preparation of the manuscript.”* We have also included the same Role of Funder statement in the cover letter at the bottom of page 2, as requested.

4. For studies involving third-party data, we encourage authors to share any data specific to their analyses that they can legally distribute. PLOS recognizes, however, that authors may be using third-party data they do not have the rights to share. When third-party data cannot be publicly shared, authors must provide all information necessary for interested researchers to apply to gain access to the data. (<https://journals.plos.org/plosone/s/data-availability#loc-acceptable-data-access-restrictions>) For any third-party data that the authors cannot legally distribute, they should include the following information in their Data Availability Statement upon submission:
1) A description of the data set and the third-party source
2) If applicable, verification of permission to use the data set
3) Confirmation of whether the authors received any special privileges in accessing the data that other researchers would not have
4) All necessary contact information others would need to apply to gain access to the data

We thank the Editor for this important clarification. In response, we have revised the **Availability of Data and Materials** statement in lines 544 to 559 to provide greater detail on the nature and governance of the study data and the process through which other researchers may request access. We confirm that there are ethical and legal restrictions on publicly sharing the underlying de-identified dataset. The dataset comprises patient-level clinical data collected from neonates included in this multicentre study. Public sharing of the de-identified dataset is restricted because the primary data are owned by third-party institutions, namely the participating hospitals and county governments, and access to and sharing of the data are subject to relevant ethical, institutional and Kenyan data governance requirements. We have additionally clarified the process for requesting access to the primary quantitative data. Researchers wishing to access the data should first contact the KEMRI-Wellcome Trust Research Programme Data Governance Committee at [dgc@kemri-wellcome.org](mailto:dgc@kemri-wellcome.org). The Committee will advise on the requirements for access and whether additional approval is required from the KEMRI Research Ethics Committee and/or the relevant facility, county government, and Ministry of Health, as appropriate to the data requested. We believe the revised statement now provides the information required to clarify the source and governance of the data and the procedure through which other researchers may seek access.

5. When completing the data availability statement of the submission form, you indicated that you will make your data available on acceptance. We strongly recommend all authors decide on a data sharing plan before acceptance, as the process can be lengthy and hold up publication timelines. Please note that, though access restrictions are acceptable now, your entire data will need to be made freely accessible if your manuscript is accepted for publication. This policy applies to all data except where public deposition would breach compliance with the protocol approved by your research ethics board. If you are unable to adhere to our open data policy, please kindly revise your statement to explain your reasoning and we will seek the editor's input on an exemption. Please be assured that, once you have provided your new statement, the assessment of your exemption will not hold up the peer review process.

We thank the editor for this clarification. As outlined in our revised “Availability of Data and Materials” statement in lines 544 to 558, the underlying data are owned and governed by the participating hospitals, counties, and Ministry of Health and cannot be made freely publicly available without the required institutional and data governance approvals. We have therefore revised our data availability statement to clearly explain these access restrictions and provide the procedure and contact details for researchers wishing to request access through the KEMRI-Wellcome Trust Research Programme Data Governance Committee. We kindly request an exemption from the requirement for unrestricted public deposition of the underlying data on this basis.

6. One of the noted authors is a group or consortium “The Clinical Information Network Group”. In addition to naming the author group, please list the individual authors and affiliations within this group in the acknowledgments section of your manuscript. Please also indicate clearly a lead author for this group along with a contact email address.

In response to the editors remarks, we have included the individual authors and their institutional affiliations for the Clinical Information Network Group in the “Acknowledgments” section (lines 568 to 660). We have also clearly indicated the lead author and their contact email address for the consortium in lines 661 to 664, as requested.

7. Your ethics statement should only appear in the Methods section of your manuscript. If your ethics statement is written in any section besides the Methods, please move it to the Methods section and delete it from any other section. Please ensure that your ethics statement is included in your manuscript, as the ethics statement entered into the online submission form will not be published alongside your manuscript.

In response to the editor’s advise, we have moved the ethics statement to the Methods section (lines 138 to 144) and removed it from other sections of the manuscript, as requested. We have also ensured that the ethics statement remains included in the manuscript and is not solely provided through the online submission form.

8. Please ensure that you refer to Figure 1 in your text as, if accepted, production will need this reference to link the reader to the figure.

In response to the reviewer’s guidance, we have cited Figure 1 in lines 295 to 297 of the revised manuscript to ensure that the figure is appropriately linked to the main text during production.

9. Please include a copy of Table 5 which you refer to in your text on page 24.

In response to the reviewer’s guidance, we have reviewed and corrected the in-text citations of the tables to ensure that all six tables are accurately cited and included in the revised manuscript. Table 5, which was previously referred to on page 24, is now included on page 23 of the revised manuscript.

10. We notice that your supplementary tables are included in the manuscript file. Please remove them and upload them with the file type 'Supporting Information'. Please ensure that each Supporting Information file has a legend listed in the manuscript after the references list.

In response to the reviewer’s guidance, we have reviewed and corrected supporting information section has the appropriate legend list consistent the journal’s styling guidelines in lines 852 to 863 and on the online submission system.

11. If the reviewer comments include a recommendation to cite specific previously published works, please review and evaluate these publications to determine whether they are relevant and should be cited. There is no requirement to cite these works unless the editor has indicated otherwise.

We thank the editor for this guidance. The reviewers did not recommend citation of any specific previously published works, and therefore no additional references were required on this basis.

**Additional Editor Comments:** Please do a substantial revisions as required.

We thank the editor for the opportunity to respond to the reviewers. We have undertaken a substantial revision of the manuscript and carefully addressed all comments and recommendations raised by the reviewers below. The revised manuscript includes changes to the presentation, methodological description, reporting, and interpretation of the study, as detailed in our point-by-point response to the comments below.

**Reviewer #1:**

The study is timely and relevant. A pragmatic, admission-based risk stratification approach using routinely recorded pSBI features aligns with clinical realities in many low-resource newborn units in Africa and Asia. The work is also well-motivated by the known heterogeneity of pSBI presentations; evidence from the old ANISA South Asia study is consistent with this heterogeneity (many pSBI episodes are not attributable to infection), while also suggesting that fatal cases with attributed causes are predominantly bacterial. This supports the plausibility that your higher-risk clusters may enrich for clinically important bacterial disease, but it also argues for caution in interpreting low-risk clusters as “overuse” or candidates for de-escalation without prospective evaluation.

We thank the reviewer for highlighting the timeliness and clinical relevance of our study, particularly the pragmatic use of routinely recorded pSBI features for admission-based risk stratification in low-resource newborn units. We appreciate the reviewer’s thoughtful contextualisation of our findings alongside evidence from the ANISA South Asia study and the important distinction between identifying higher-risk clusters and interpreting lower-risk clusters. We agree that the latter should not be interpreted as evidence of non-infectious illness or as justification for antibiotic de-escalation without prospective evaluation, and we have taken this caution into account in revising the manuscript in lines 481 to 491 of the discussion section.

I found some substantive issues

1) Your Primary result inconsistency: four vs five clusters (must fix throughout)
In the Discussion you state “four clinically distinct clusters” and list minimal, moderate, substantial and critical (lines 416–418), but later state “our findings support five distinct neonatal sepsis clusters” (line 457). The Conclusions again state “four clinically distinct clusters” (lines 513–515). Earlier sections and tables (as presented in the submission materials provided for review) indicate five clusters (minimal, low, moderate, substantial, critical). Please correct this across all sections, tables and figure labels; as written, this undermines confidence in the core finding.
Specific edits: change “four” → “five” at line 416 and lines 513–515, and include “low risk” in the cluster list at lines 416–418.

We thank the reviewer for identifying this inconsistency. This was a wording error in the “Discussion” and “Conclusions” rather than a discrepancy in the underlying analysis, which consistently identified five clusters: minimal, low, moderate, substantial, and critical risk. We have corrected the affected text throughout the revised manuscript (e.g. lines 426 to 429, 521 to 524), including changing “four” to “five” and explicitly including the low-risk cluster in the cluster descriptions, and have checked the tables and figure labels to ensure that the five-cluster solution is reported consistently throughout.

2) Clarify what is meant by “external validation” (wording and reporting)
I may be misunderstanding the intended validation framework, but “external validation” reads as if it refers both to applying the fitted LCA model in new hospitals and to validating a separate parsimonious assignment model against LCA-derived labels?. Could you clarify the type and extent of validation? A statistician may help to clarify this better.

We agree with the review that “external validation” was conflated with transportability concept (i.e. once you have identified the five clusters using LCA, can you develop a simpler, clinically usable classifier that accurately assigns subsequent patients to those predefined clusters?). We have therefore amended lines 119 to 125 of the objectives to clarify this important distinction, with objective 2(a) addressing external validity (i.e. *whether* *the clusters themselves replicate/transfer to new hospitals)* and 2(b) addressing transportability (i.e. *whether once you have identified the clusters using LCA, can you develop a simpler, clinically usable classifier that accurately assigns subsequent patients to those predefined clusters).*

We have also updated lines 241 to 258 of the methods section to better clarify the different approaches to model external validation and transportability that the revised objective 2 addresses. In the in the results section of revised manuscript, table 4 and lines 361 to 369 address the external validation objective (2a) with tables 5 and 6 and lines 373 to 386 now addressing objective 2(b).

3) Reduced-feature claim does not match the predictors used
In the Discussion you state that “a subset of 11 pSBI signs and symptoms were sufficient to determine cluster membership” (lines 421–425). However, the reduced assignment model presented in the submission materials includes non-sign physiological predictors (birth weight, gestational age, early-onset age) - otherwise known as risk factors. If these variables are part of the intended bedside tool, please align the wording and justify inclusion; if the intent is a “signs-only” tool, refit the reduced model accordingly and report that version. N. Connor et al. stratified these risk factors in various domains for bacterial pbsi here: <https://gh.bmj.com/content/bmjgh/7/11/e009706.full.pdf> for reference.

We thank the reviewer for identifying this important clarification. We agree that our previous wording incorrectly implied that the reduced assignment model relied exclusively on pSBI signs and symptoms. The intention was to describe the reduced set of predictors required for cluster assignment, which comprised 11 pSBI signs and symptoms together with birth weight, gestational age, and age at early onset. We have therefore revised the text to explicitly distinguish the clinical signs and symptoms from these additional risk factors and to accurately describe the predictors used in the reduced assignment model (revised manuscript, lines 432 to 436).

4) WHO comparator operationalisation needs to be transparent; avoid “gold standard” framing Supplementary Table 1 provides WHO pSBI management categories, but the manuscript should clearly describe how recorded variables were mapped to WHO criteria (particularly feeding variables, cyanosis/hypoxia, chest indrawing, and “fast breathing only”). Where the manuscript reports any unclassifiable cases, please explain why (e.g., signs not represented in WHO table vs missingness preventing assignment). Also, WHO is best described as a guideline-based severity/management classification rather than a “gold standard.”

We thank the reviewer for this clarification. We have clarified in lines 154 to 159 that the hospital admission forms were designed to capture clinical variables in alignment with WHO and national clinical guidelines, and the recorded variables therefore correspond directly to the WHO pSBI criteria without requiring additional clinical interpretation or ad hoc mapping. We have also replaced all the “gold standard” terminology with WHO guideline-based severity/management classification, as recommended in lines 126 to 128 and 269 to 271.

5) Antibiotic stewardship implications are over-extended for the current evidence
The Discussion suggests that antibiotic use in minimal/low risk clusters “might be indicative of antibiotic overuse or misuse” and speculates about less aggressive strategies (shorter IV, oral antibiotics) (lines 473–479). Given pSBI heterogeneity (including ANISA’s earlier findings in South Asia - that many pSBI episodes are not attributable to infection), it is reasonable to motivate stewardship evaluation, but not to imply inappropriate use or propose de-escalation based on retrospective signs-only clusters without safety/efficacy testing. Consider reframing these points as hypotheses for prospective evaluation rather than clinical implications of the present analysis.

We agree with the reviewer on this important point that our retrospective analysis cannot determine whether antibiotic use in the minimal and low mortality-risk clusters was inappropriate, nor can it support recommendations for antibiotic de-escalation. We have therefore revised the discussion in lines 484 to 491 to state that this warrants further evaluation of whether antibiotic use could be safely reduced in some patients, rather than being interpreted as evidence of irrational antibiotic use. We now frame the potential for different antibiotic treatment strategies, including shorter intravenous courses or, where clinically appropriate, oral therapy, as hypotheses for prospective evaluation of safety and efficacy, rather than as clinical implications of the present study.

Minor issues / specific edits

Line 413–414: remove duplicated wording “inform to inform.”

Line 431–432: “low gestation age” → “low gestational age.”

Line 433: “on the hand” → “on the other hand.”

We thank the reviewer for identifying these typographical and wording errors. We have addressed all three comments in the revised manuscript by correcting the duplicated wording (line 425) changing “low gestation age” to “low gestational age” (line 441) and correcting “on the hand” to “on the other hand.” (line 443)

Supp. Table 1: add a brief note that WHO guidance spans 0–59 days but this analysis is restricted to 0–28 days.

In response to the reviewer’s comment, we have added a brief note at the bottom of S1 Table in lines 853 to 855 to explain that while WHO guidance spans 0 to 59 days of life, our this analysis is restricted to 0 to 28 days (i.e. neonatal period).

Reviewer #2: This is an interesting and potentially valuable study using a large multicentre dataset. Given the substantial statistical and machine-learning components of this study, I recommend that the manuscript also be reviewed by a statistical/machine-learning modelling expert.

We thank the reviewer for recognising the interest and potential value of the study and for highlighting the importance of the statistical and machine-learning components. We appreciate the recommendation for additional statistical/machine-learning expertise and have carefully reviewed and strengthened the methodological and analytical aspects of the manuscript accordingly.

Comments
-The research gap could be more explicitly explained particularly how this study addresses limitations in the existing literature.

We thank the reviewer for this helpful comment. We agree that the original background described limitations of existing neonatal sepsis phenotyping approaches but did not sufficiently articulate the specific research gaps addressed by our study. We have added lines 104 to 111 in the revised manuscript to clarify that existing approaches often rely on laboratory or biomarker variables unavailable at admission in many SSA settings, and that the generalisability of data-driven phenotypes across clinical settings requires evaluation. We now explicitly state that our study addresses these gaps by: (1) identifying neonatal risk phenotypes using routinely collected pSBI signs; (2) assessing reproducibility in independent hospital populations; (3) evaluating a parsimonious classifier for assigning new patients to predefined classes; and (4) comparing the prognostic value of these clusters with the WHO classification. These revisions clarify how our study advances beyond phenotype derivation to assess reproducibility, transportability, and clinical applicability.

-Avoid overly strong wording e.g. “largely ungeneralisable”

In response to the reviewer’s comment, we have revised the text in lines 103 to 107 to use more measured language, noting that findings from studies incorporating such variables, and the resulting clusters, may have limited applicability to many SSA hospital settings.

-Is it appropriate to describe the WHO neonatal sepsis severity classification as the “gold standard”? As classifications may evolve over time, simply referring to it as the “WHO classification” may be more appropriate.

In response to the reviewer’s request, we have replaced all the “gold standard” terminology with WHO guideline-based severity/management classification in lines 126 to 128 and 269 to 271, as recommended.

-Justify the use of hospital volume to define the development and validation datasets, as differences in hospital size may reflect differences in patient populations and care settings.

In response to the reviewer’s point, we have clarified in the revised manuscript in lines 169 to 174 that, although hospital volume may capture differences in patient populations and care settings, it was used as a pragmatic criterion to define the development and validation datasets. This ensured that the development dataset contained a sufficiently large number of neonates to support stable estimation of the model-based clusters while minimising the influence of very small hospital-specific samples.

-Provide a baseline comparison of the development and validation populations to assess potential differences.

In line with the recommendations by the reviewer, we have added table 1 to provide a baseline comparison of the patient characteristics across the different datasets. Together with figures S1 Fig and S2 Fig, these allow the reader to assess any potential differences in the populations in the different datasets.

-Please clarify how the labels “minimal,” “low,” “moderate,” “substantial,” and “critical” risk were defined and assigned to the clusters, including whether any predefined mortality thresholds were used.

We thank the reviewer for requesting this clarification. We have updated lines 219 to 222 of the methods section in the revised manuscript to clarify that the cluster labels were assigned by the research team after examining the mortality outcome and the characteristic pattern of conditional response probabilities across the observed clinical variables for each LCA-derived cluster. We have also added lines 341 to 343 in the results section to explain that the labels “minimal,” “low,” “moderate,” “substantial,” and “critical” were intended as clinically meaningful descriptors of the relative mortality risk across the clusters and that no predefined mortality thresholds were used to assign these labels.

-Avoid suggesting that antibiotic use in lower-risk clusters represents overuse or misuse as appropriateness of antibiotic treatment was not assessed.

We agree with the reviewer on this important point that our retrospective analysis cannot determine whether antibiotic use in the minimal and low mortality-risk clusters was inappropriate, nor can it support recommendations for antibiotic de-escalation. We have therefore revised the discussion in lines 484 to 491 to state that this warrants further evaluation of whether antibiotic use could be safely reduced in some patients, rather than being interpreted as evidence of irrational antibiotic use. We now frame the potential for different antibiotic treatment strategies, including shorter intravenous courses or, where clinically appropriate, oral therapy, as hypotheses for prospective evaluation of safety and efficacy, rather than as clinical implications of the present study.

-Treatment received may have influenced mortality across the clusters.

We agree with the reviewer that the mortality may also have been influenced by differences in treatment and other aspects of care received during hospitalisation, which were not accounted for in the clustering model. We have added lines 475 to 478 to highlight this, while also arguing that the observed differences in mortality between clusters should not be interpreted as reflecting differences in underlying disease severity alone, and prospective studies are needed to disentangle the contribution of baseline phenotype from subsequent treatment and care.

-Discuss the exclusion of out-born neonates as a limitation.

We thank the reviewer for raising this critical point. On review of the manuscript, we identified an inadvertent inconsistency in the description of the study population and eligibility criteria rather than an actual exclusion of out-born neonates from the analysis. We have therefore removed the statement restricting the analysis to in-born neonates from the “Abstract” section (line 36) and removed the corresponding exclusion criterion for out-born neonates from the “Study Design and Participants” section of the Methods. The revised description now accurately reflects the study population, which included both in-born and out-born neonates, as also shown in Figure 1. As out-born neonates were therefore included in the analysis, we have not added their exclusion as a limitation in the Discussion.

-Temper the generalisability to SSA, as the study was conducted only in Kenya.

We thank the reviewer for this important point. We have revised the manuscript to more appropriately qualify the potential applicability of our findings beyond Kenya. Specifically, we clarify in the Methods (lines 149 to 159) that the participating hospitals use structured clinical documentation aligned with WHO and national clinical guidelines, and that this approach to clinical form design and data collection in routine clinical settings is also used across East and Southern Africa [1, 2]. We also note that the participating CIN hospitals form part of the broader NEST360 multi-country programme, with NEST360 implemented in a subset of hospitals already engaged in CIN and supported by CIN’s routine data and quality-improvement activities. We have updated the discussion in lines 496 to 500 and 511 to 514, to refer specifically to the potential applicability of the clusters to similar SSA hospital settings, rather than implying generalisability across SSA as a whole [1, 2].

**References**

1.	Murless-Collins, S., et al., *Blood culture versus antibiotic use for neonatal inpatients in 61 hospitals implementing with the NEST360 Alliance in Kenya, Malawi, Nigeria, and Tanzania: a cross-sectional study.* BMC pediatrics, 2023. **23**(Suppl 2): p. 568.

2.	Cross, J.H., et al., *Neonatal inpatient dataset for small and sick newborn care in low-and middle-income countries: systematic development and multi-country operationalisation with NEST360.* BMC pediatrics, 2023. **23**(Suppl 2): p. 567.
